# Mononuclear phagocyte–specific cGAS/STING targeting suppresses experimental choroidal neovascularization

**DOI:** 10.1172/jci.insight.203452

**Published:** 2026-08-10

**Authors:** Le Shi, Durgadas Cherukaraveedu, Hongkwan Cho, Kaoru Ri, Lingli Zhou, Yingxue Cao, Narendra Kale, Zhenhua Xu, Wathsala Liyanage, Rangaramanujam M. Kannan, Elia J. Duh

**Affiliations:** 1 Department of Ophthalmology, School of Medicine, Johns Hopkins University, Baltimore, Maryland, USA.; 2 Center for Nanomedicine at the Wilmer Eye Institute, Johns Hopkins University School of Medicine, Baltimore, Maryland, USA.; 3 Department of Chemical and Biomolecular Engineering, Johns Hopkins University, Baltimore, Maryland, USA.

**Keywords:** Inflammation, Ophthalmology, Vascular biology, Drug therapy, Macrophages

## Abstract

Neovascular age-related macular degeneration (nAMD) is a major cause of blindness and is characterized by pathologic angiogenesis, specifically choroidal neovascularization (CNV). Mononuclear phagocytes (MPs), including infiltrating systemic monocyte-derived macrophages and retinal microglia, play critical roles in promoting CNV. The cGAS/STING pathway is increasingly implicated in multiple neuronal and systemic diseases and recently in ocular neovascularization. Given its roles across multiple cell types and the absence of MP-targeted therapies, we investigated the MP-specific role of cGAS/STING and a strategy for its selective targeting. In the laser-induced CNV mouse model, cGAS/STING was predominantly expressed in MPs. To enable selective targeting, we used a hydroxyl dendrimer (HD) previously shown to target MPs. HD conjugated to Cy3 selectively localized to MPs in laser CNV. HD conjugated to the STING inhibitor SN-011 (HD-SN-011) effectively inhibited cGAS/STING activation in cultured MPs. In the laser-CNV model, HD-SN-011 significantly reduced CNV leakage and lesion size, both important clinical endpoints in nAMD. RiboTag profiling confirmed selective suppression of cGAS/STING signaling and inflammatory gene expression in MPs. Together, our results implicate the specific importance of MP cGAS/STING signaling in CNV and provide proof of concept for specific modulation of STING in MPs as a therapy for nAMD.

## Abstract 2

A dendrimer nanoparticle-based STING inhibitor targeting mononuclear phagocytes reduces retinal inflammation and abnormal blood vessel growth.

## Graphical abstract

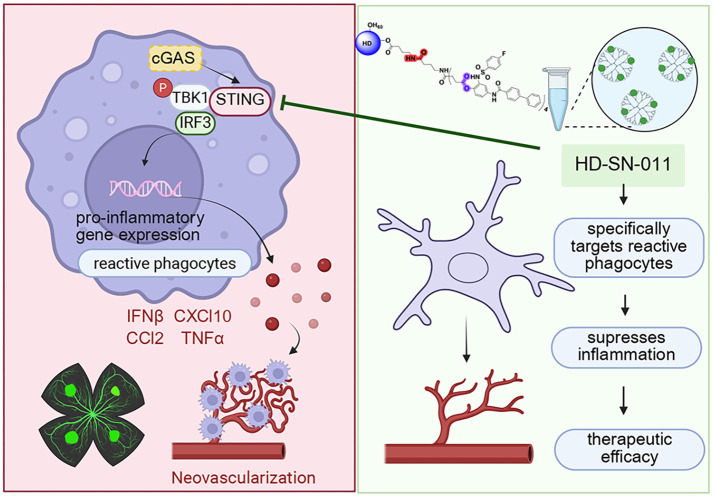

## Introduction

Age-related macular degeneration (AMD) is the leading cause of legal blindness in developed countries, and its impact is increasing with the aging population (1, 2). Earlier stages of AMD are characterized by a combination of aging changes, chronic inflammation, oxidative stress, and lipid and lipoprotein deposition that damage the outer retina, underlying retinal pigment epithelium, and choroid (1). Ultimately, the inflammatory and degenerative milieu beneath the retina promotes pathologic choroidal neovascularization (CNV), the hallmark of neovascular AMD (nAMD), which accounts for 90% of blindness in AMD. Regular intravitreal injections of anti-VEGF drugs are the current therapy for nAMD. However, incomplete response to anti-VEGF therapy, with persistent disease activity, continues to be a major unmet need (3). Consequently, continued visual loss remains a major issue.

Chronic inflammation and abnormal activation of the innate immune system are increasingly appreciated to play crucial roles in the pathogenesis of AMD (4). Both earlier stages of AMD and nAMD extensively involve myeloid cells, specifically mononuclear phagocytes (MPs), including retinal microglia, choroidal macrophages, and monocyte-derived macrophages recruited from the circulation (5, 6).

Activated MPs accumulate around Bruch’s membrane and CNV lesions, creating a persistent inflammatory microenvironment that promotes angiogenesis and tissue remodeling (7, 8). Current literature suggests an important role for both macrophages and microglia in the regulation of CNV, with the state of these cell types being an important determinant of their pathogenicity. Macrophages are present in surgical CNV membranes from patients with nAMD (9). Systemic depletion of macrophages or microglia, or reduced recruitment of systemic monocytes to tissue beds, suppresses CNV (6, 7, 10–14). Notably, MP state can have a profound impact on regulation of CNV, with proinflammatory macrophages being particularly pathogenic in CNV (15). The modulation of MP-derived neuroinflammation has therefore emerged as a potentially important therapeutic strategy for treatment of CNV in wet AMD, with the objective of pharmacologic modulation of MPs away from pathogenic states. In this regard, there is a great need for identification of critical pathways that regulate pathogenic activation of MPs.

cGAS/STING signaling is increasingly recognized as important in neurodegenerative, neuroinflammatory, and vascular diseases, with potentially important effects in multiple cell types, including innate immune cells (16). Upon detection of cytosolic DNA, cGAS catalyses synthesis of cyclic GAMP (cGAMP), which activates STING and induces downstream phosphorylation of TBK1 and IRF3. This process leads to the production of type I interferons and proinflammatory cytokines (17, 18). cGAS/STING plays a central role in multiple neurodegenerative diseases, including Alzheimer, Parkinson, Huntington, and ALS (16, 19). Notably, emerging evidence suggests a role for cGAS/STING signaling in driving CNV (20, 21), although the cellular context for this signaling has not been definitely established, especially important with the multicellular nature of CNV. In the laser-induced CNV model, the cGAS/STING pathway is upregulated, and treatment with pharmacologic STING inhibitors such as H151 or C176 reduces inflammation and neovascular lesion size. Even though cGAS/STING modulation could be a promising treatment for CNV and many neuronal and systemic conditions, cell-specific targeting of cGAS/STING is highly desirable to avoid adverse effects, including reduction in tumor surveillance and greater susceptibility to infection (19, 22–24). It is therefore critical to identify the cell type(s) in which cGAS/STING signaling is particularly pathogenic in CNV, a disease process that involves multiple cell types, including those from the retinal pigment epithelium (RPE), endothelial cells, immune cells, neuronal cells. This objective can be challenging given the relevance of cGAS/STING in a variety of cells. For instance, microglial cGAS/STING has been implicated in aging-related inflammation and neurodegeneration (25). Endothelial cell cGAS/STING has been implicated in a model of endotoxemia-induced shock (26). Neuronal STING plays a critical pathogenic role in inflammation-induced neurodegeneration in multiple sclerosis (27).

Given the multicellular nature of CNV and the established role of MPs in CNV, approaches to target cGAS/STING inhibitors to MPs can be valuable.

In this regard, polyamidoamine (PAMAM) hydroxyl dendrimers (HDs) provide an ideal approach, both in offering a means to specifically target “reactive” MPs in disease pathogenesis and in providing an approach for specific therapeutic modulation of these cells (28). Importantly, these HDs and their drug conjugates exhibit a high selectivity in targeting reactive MPs in both the brain (29) and retina (30), without a need for targeting ligands. This targeting has been validated in more than 50 models in 6 species, and the “biophysical mechanism” of pathology-dependent uptake has been described previously, likely due to the high phagocytic activity of these cells with internalization (enhanced cellular uptake of the HD and its drug cargo; refs. 28, 31). Our previous research has demonstrated HDs to be efficient nanosystems for effective and selective delivery of diverse therapeutic cargos (28). Among these, OP-101 (HD conjugated with *N*-acetyl cysteine [NAC]) demonstrated promising safety and efficacy in a phase IIa clinical trial in hospitalized severe COVID-19, upon intravenous administration. The systemic treatment improved survival (>40% over standard of care), reducing COVID-19 measures, and significantly reducing blood biomarkers of neurological injury correlated with long COVID-19 (32, 33). Notably, HD-conjugated drugs have been successfully used for modulation of MPs in experimental models of eye disease, including nAMD (34), ischemic retinopathy (30), and glaucoma (35). A phase IIa trial for once-a-month subcutaneous HD-conjugated sunitinib analog (migaldendranib) showed greater than 80% reduction in the need for intravitreal anti-VEGF injections in wet AMD and diabetic macular edema, while helping both eyes, illustrating the safety and “back of the eye” delivery of systemic HD-drug conjugates. Chronic administration of the conjugate over 10 months did not cause any toxicity issues in these patients (36). These studies collectively highlight the versatility of HD as a clinically translatable platform for targeted and sustained ocular drug delivery.

With their specific targeting of reactive MPs, HDs provide the capability both for mechanistic interrogation and therapeutic modulation of these immune cells. In the current study, we found robust colocalization of cGAS with the mononuclear phagocyte marker ionized calcium–binding adapter molecule 1 (IBA1) within neovascular lesions in the laser-induced CNV mouse model. This prompted us to determine the functional importance of cGAS/STING signaling in MPs in this setting. To achieve selective targeting of this pathway in activated phagocytes, we synthesized the conjugate HD-SN-011, consisting of an HD conjugated to STING inhibitor SN-011 (37). Using HD-Cy3 as a fluorescent tracer, we confirmed that HD-Cy3 was efficiently taken up by MPs in both cell culture and in the laser-induced CNV model, with fluorescence signals specifically localized to activated MPs in the CNV region. Strikingly, HD-SN-011 effectively inhibited activation of the cGAS/STING signaling pathway in MPs in vitro and in vivo, and significantly attenuated laser-induced CNV. Together, this demonstrates the specific importance of MP cGAS/STING signaling in CNV and highlights the use of an HD-conjugated STING inhibitor for specific modulation of MPs for nAMD and potentially other inflammatory diseases of the retina and CNS.

## Results

### The cGAS/STING pathway is active in MPs in both human and mouse nAMD.

**Figure 1:**
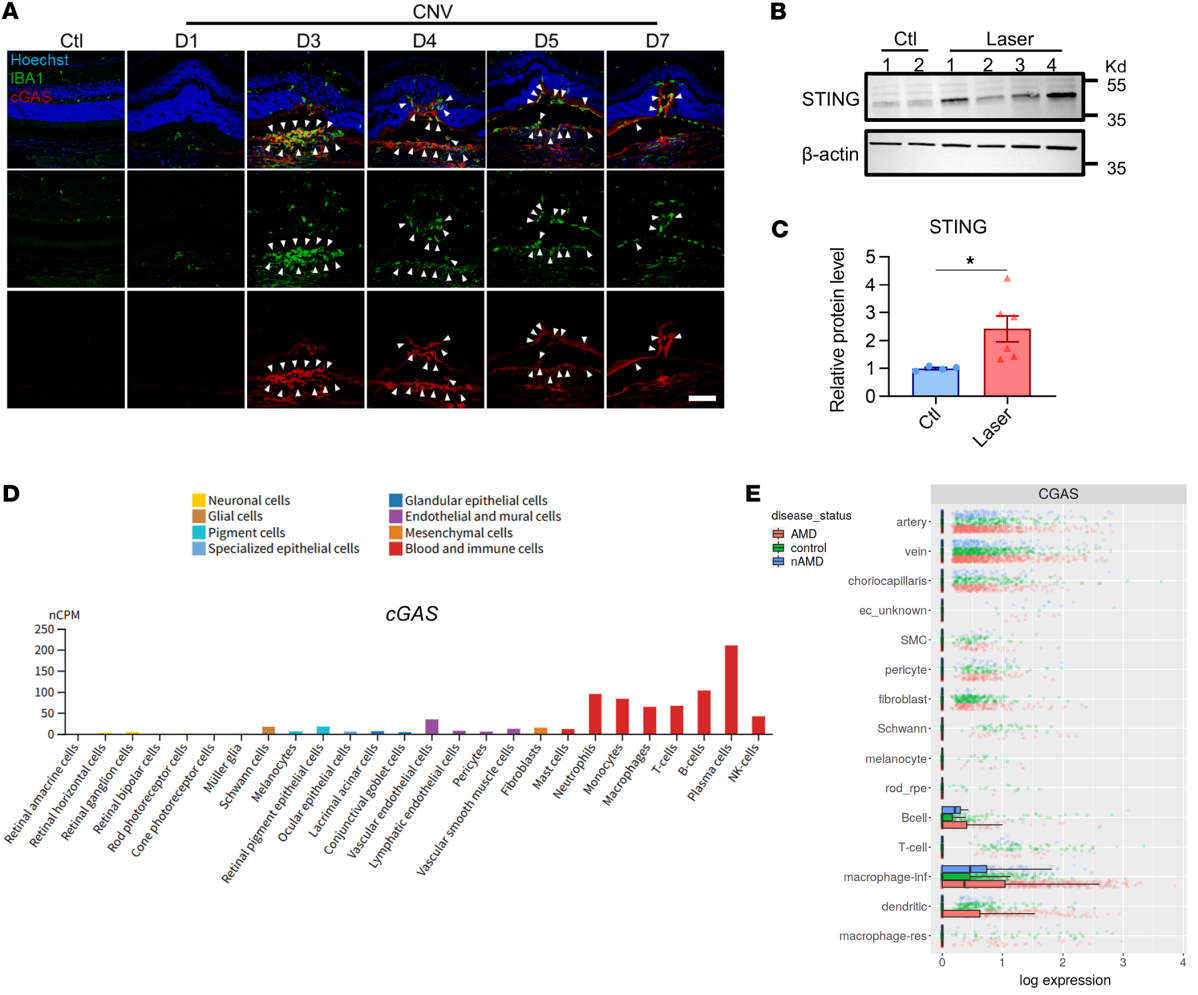
The cGAS/STING pathway is active in microglia/macrophages in human nAMD and laser-induced CNV mice. (**A**) Representative immunofluorescence images of ocular cross sections from control (Ctl) and experimental mice on day 1 (D1), D3, D4, D5, and D7 after laser induction of CNV. Sections were stained for IBA1 (green), cGAS (red), and Hoechst (blue). Arrowheads indicate IBA1^+^cGAS^+^ double-positive cells localized within or surrounding the CNV lesions. Scale bar: 100 μm. (**B**) Western blot analysis of STING in retina-RPE-choroid complexes from control and laser CNV eyes on D3 (*n* = 4–6 per group). β-Actin was used as a loading control. (**C**) Quantitation of STING protein levels in **B**. (**D**) cGAS expression levels across human ocular cell populations based on single-cell RNA-seq data obtained from the Human Protein Atlas. (**E**) Dot plot of single-cell RNA-seq expression of *cGAS* across retinal and choroidal cell populations from human donor eyes with AMD, nAMD, and controls. Individual dots represent single cells. Bar graphs depict the mean ± SEM. Note that the mean for most cell populations is close to zero. Statistical significance was assessed by 2-sided, unpaired *t* test. *P < 0.05.

To determine whether the cGAS/STING pathway is active in nAMD and to identify the responsible cell type, we examined the temporal dynamics of cGAS/STING signaling in a laser-induced CNV mouse model. Consistent with previous reports (6), in retinal cross sections, IBA1^+^ MPs were present in the CNV lesion area as early as day 1 after laser injury, peaked on day 3, and gradually declined thereafter, whereas in controls, IBA1^+^ cells remained confined to the inner retina in cross section (Figure 1A). Notably, cGAS was highly colocalized with reactive IBA1^+^ MPs within the CNV lesions but absent in IBA1^+^ cells located outside the lesion, indicating that cGAS expression is primarily associated with lesion-resident MPs (Figure 1A). We also performed RPE/choroid flat-mount staining, which similarly showed that mild cGAS signal began to appear at day 1 after laser, without obvious cGAS^+^IBA1^+^ cells. At days 3 and 4, IBA1^+^ cells markedly accumulated within the lesion, and abundant colocalization signals of cGAS and IBA1 were observed; by days 5 and 7, the cGAS signal and colocalization gradually decreased (Supplemental Figure 1; supplemental material available online with this article; https://doi.org/10.1172/jci.insight.203452DS1). Western blot analysis further showed a marked increase in STING protein levels in the retina-choroid-RPE complex at day 3 after laser administration compared with controls (Figure 1, B and C). We were interested in deducing the human ocular cell types that endogenously express cGAS. For this purpose, we first analyzed single-cell transcriptomic data from the Human Protein Atlas eye database. The results showed that cGAS expression was particularly prominent in immune cell populations, including macrophages and lymphocytes, although neurons, glial cells, epithelial cells, and vascular-associated cells exhibited expression at lower levels (Figure 1D). We next analyzed single-cell RNA-sequencing (RNA-seq)data from human retinal samples obtained from nAMD, dry AMD, and control donors. This analysis further confirmed that cGAS expression was particularly prominent in immune cells, with much lower expression in non-immune cell populations (Figure 1E). Among immune subsets, inflammatory macrophages exhibited the highest cGAS expression. Moreover, cGAS expression in inflammatory macrophages was substantially upregulated in both nAMD and dry AMD samples compared with controls (Figure 1E). Collectively, these results demonstrate that the cGAS/STING pathway is strongly active in reactive MPs in both human nAMD and the experimental laser-induced CNV model, suggesting the potential involvement of this pathway in the regulation of CNV by reactive MPs.

### Synthesis, characterization, and in vitro drug release profile of the HD-SN-011 conjugate.

**Figure 2:**
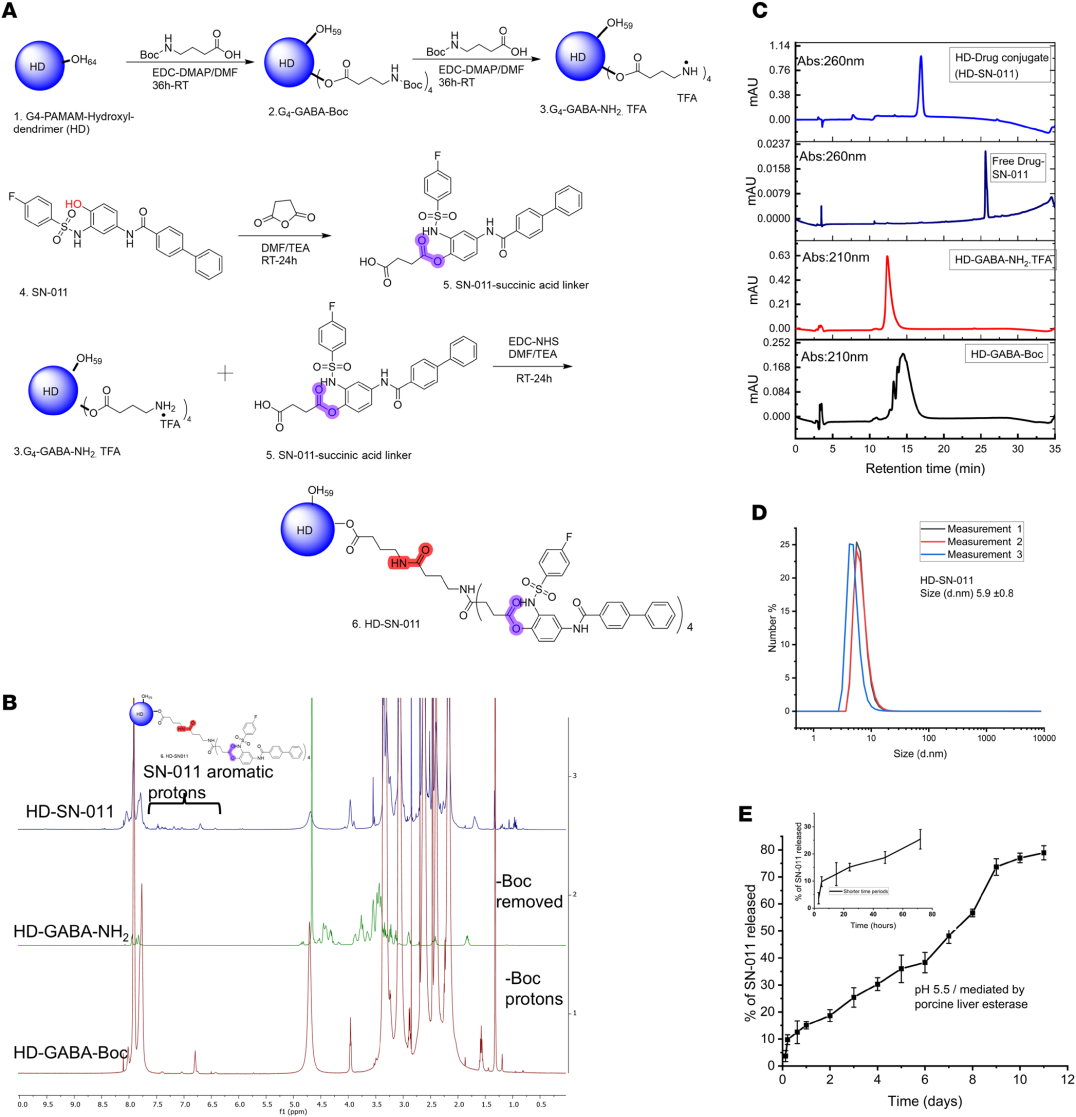
Synthesis and characterization of HD-SN-011 with improved solubility and physicochemical stability. (**A**) Schematic showing the multistep chemical synthesis of the HD-SN-011 conjugate. (**B**) ^1^H-NMR confirmed the formation of the HD-SN-011 conjugate via amide bonds on HD. (**C**) Analytical HPLC confirmed the conjugation of the GABA linker to HD at 210 nm detection (specific for HD), and the HD-drug conjugation at 260 nm detection. HD-SN-011 showed over 98% purity with a distinct retention time of approximately 17 minutes compared with the free drug at 26 minutes and HD-GABA-NH2 at 12.5 minutes. (**D**) DLS studies of the HD-SN-011 conjugate confirmed the monodisperse particle distribution with a size of approximately 5.9 nm. (**E**) The HD-SN-011 conjugate showed ester-mediated release of pure SN-011 under acidic pH (0.5 M citrate buffer). The analytical HPLC results show that nearly 75% of the drug was released over 11 days.

Our previous studies demonstrated that dendrimer-drug conjugates enable selective targeting of reactive MPs (28, 32, 34). To specifically modulate the cGAS/STING pathway in reactive phagocytes, we developed an HD conjugate of SN-011 (HD-SN-011) (Figure 2A). The intermediate compound HD-GABA-Boc (compound 3) was synthesized via ester coupling between HD and Boc-GABA-OH and characterized by proton nuclear magnetic resonance (¹H-NMR). The spectrum indicated approximately 4 GABA linkers conjugated to the HD backbone, based on the integration ratio between the *tert*-butyl protons of Boc-GABA-OH (1.35 ppm) and the amide protons of HD in the aromatic region. After Boc deprotection using 25% trifluoroacetic acid (TFA) in dichloromethane (DCM) under ice-cold conditions, the resulting HD-GABA-NH_2_ was obtained for eventual conjugation with modified SN-011. SN-011 was modified via an ester bond using succinic anhydride as a linker molecule. The final compound HD-SN-011 was synthesized by coupling the acid-functionalized SN-011 (SN-011 succinic acid linker) and HD-GABA-NH_2_ via an amide bond using EDC/NHS chemistry. The final HD-SN-011 conjugate was characterized by ^1^H-NMR and confirmed approximately 4 SN-011 molecules per dendrimer, with aromatic proton peaks (6.5–7.9 ppm) corresponding to SN-011 (Figure 2B). The ester linkage between SN-011 and the succinic acid spacer was designed to be hydrolyzable for intracellular drug release. Analytical high-performance liquid chromatography (HPLC) revealed a single dominant peak with greater than 98% purity for the HD-SN-011 conjugate (Figure 2C). Dynamic light scattering (DLS) showed that HD-SN-011 was monodisperse, with an average hydrodynamic diameter of 5.92 ± 0.88 nm and a ζ potential of –8.34 ± 0.21 mV, indicating excellent stability (Figure 2D). In vitro drug-release assays demonstrated that HD-SN-011 exhibited a sustained and esterase-responsive release profile (Figure 2E). Drug release was performed in 0.5 M citrate buffer (pH 5.5) containing porcine liver esterase, which mimics lysosomal conditions. The release of free SN-011 was quantified by HPLC at 260 nm based on the area under the curve (AUC). The enzyme was replenished every 48 hours to maintain catalytic activity, and samples were collected at predefined time points. Approximately 50% of SN-011 was released within 7 days and approximately 75% within 12 days, indicating controlled hydrolytic cleavage of the ester linkage under lysosome-like conditions. Collectively, these results demonstrate that HD-SN-011 is stable and “monodisperse,” exhibiting sustained and enzyme-responsive drug release under lysosome-mimicking conditions, thereby providing a basis for its further evaluation in ocular delivery applications.

### HD-SN-011 markedly reduces the in vitro and in vivo cytotoxicity of free SN-011.

**Figure 3:**
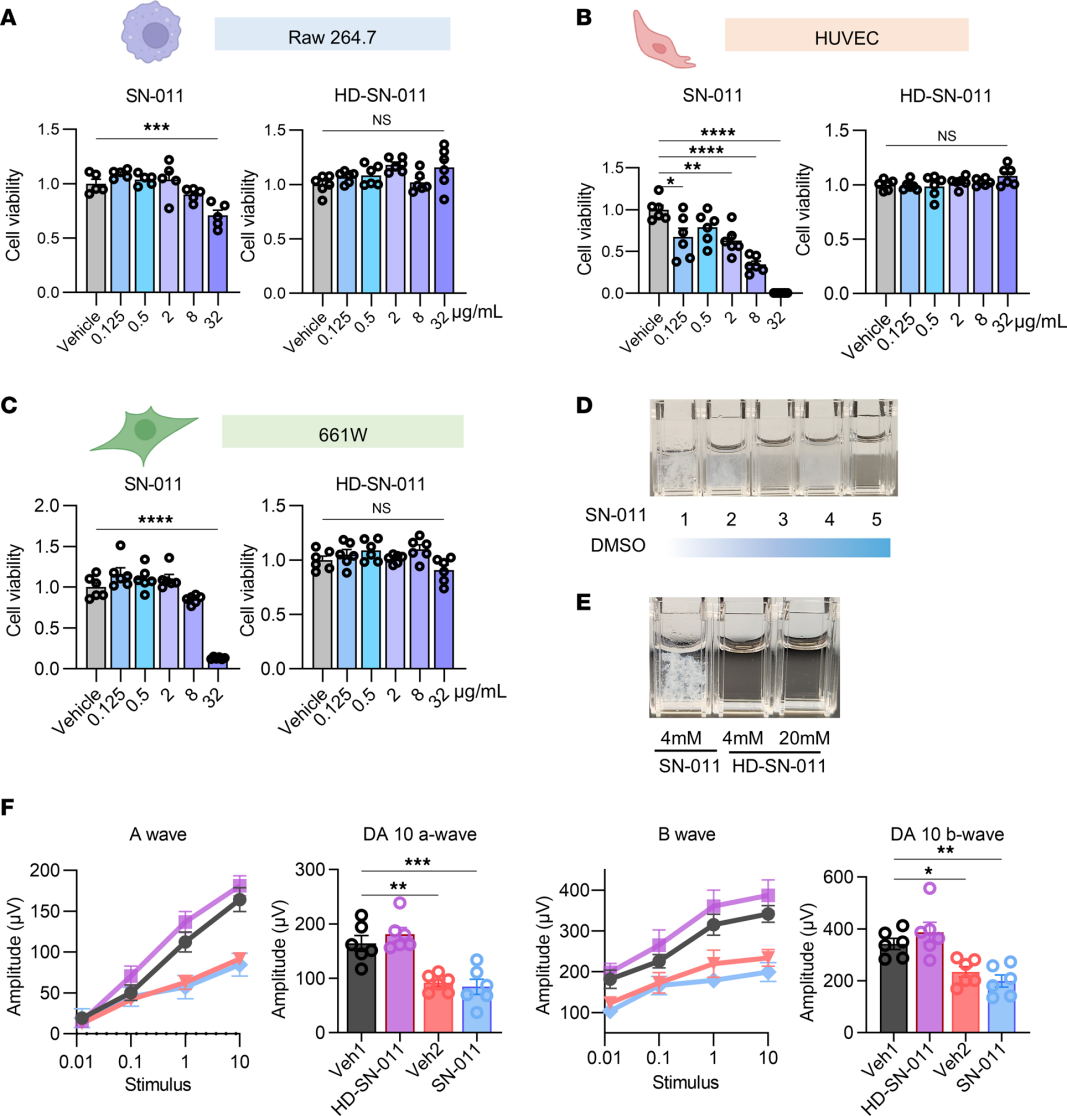
HD-SN-011 exhibits solubility, low cytotoxicity, and excellent ocular safety. (**A**–**C**) Viability assays in RAW264.7 cells (**A**), HUVECs (**B**), and 661W cells (**C**). Cells were treated with vehicle, free SN-011, or HD-SN-011 at the indicated concentrations for 16 hours, followed by measurement of cell viability using the CTG assay. A concentration of 32 μg/mL corresponds to 69 μM SN-011. (**D**) Representative images showing the solubility of free SN-011 in various solvent formulations. From left to right, the solvents are (1) 1% DMSO + 99% saline, (2) 3% DMSO + 10% PEG300 + 3% Tween 80 + 84% saline, (3) 5% DMSO + 40% PEG300 + 5% Tween 80 + 50% saline, (4) 8% DMSO + 40% PEG300 + 5% Tween 80 + 47% saline, and (5) 10% DMSO + 40% PEG300 + 5% Tween 80 + 45% saline. (**E**) Representative images showing the solubility of free SN-011 and HD-SN-011 at different concentrations when dissolved in saline. A concentration of 4 mM corresponds to 1.85 mg/mL SN-011, and 20 mM corresponds to 9.25 mg/mL SN-011. (**F**) In vivo retinal safety was assessed by ERG after intravitreal injection. Four experimental groups were included: vehicle control 1 (saline), HD-SN-011 dissolved in saline, vehicle control 2 (10% DMSO + 40% PEG300 + 5% Tween 80 + 45% saline), and free SN-011 dissolved in vehicle 2. Scotopic A- and B-wave amplitudes were recorded in response to increasing flash intensities at 7 days after injection. Data are represented as mean ± SEM. Statistical significance was assessed by 1-way ANOVA with Šídák’s multiple-comparison test. NS, not significant. *P < 0.05, **P < 0.01, ***P < 0.001, ****P < 0.0001.

We next evaluated the biosafety of HD-SN-011 in multiple cell types, including murine macrophage-like RAW264.7 cells, human umbilical vein endothelial cells (HUVECs), and mouse photoreceptor-derived 661W cells. The results showed that free SN-011 induced significant cytotoxicity in all 3 cell types when the concentration exceeded 8 μg/mL, whereas cells treated with HD-SN-011 maintained more than 90% viability even at higher concentrations up to 32 μg/mL of conjugated SN-011, exhibiting negligible cytotoxicity (Figure 3, A–C). Notably, HUVECs were particularly sensitive to free SN-011, showing a marked decrease in viability at 0.125 μg/mL with a clear dose-dependent trend (Figure 3B). These findings indicate that conjugation to the HD effectively mitigates the concentration-dependent cytotoxicity of SN-011 (by greater than 250-fold) and improves its biocompatibility.

Previous studies reported that intravitreal injection of SN-011 at 4 mM effectively reduced laser-induced CNV (20). Based on this, we evaluated the potential retinal toxicity of 4 mM HD-SN-011 and free SN-011 following intravitreal injection. Because free SN-011 is highly hydrophobic and poorly soluble in water, we first tested several solvent systems containing various ratios of DMSO, PEG300, Tween 80, and saline. Formulations with low DMSO content (1%–8%) or lacking PEG300 and Tween 80 appeared turbid or showed visible precipitation, whereas only the mixture containing 10% DMSO, 40% PEG300, 5% Tween 80, and 45% saline formed a clear solution (Figure 3D). Therefore, this formulation was used as the vehicle for free SN-011. In contrast, HD-SN-011, owing to the hydrophilic dendrimer carrier, was completely soluble in saline at both 4 mM and 20 mM without visible precipitation (Figure 3E).

To assess in vivo ocular safety, we performed electroretinography (ERG) at 7 days after intravitreal injection of different drug formulations in mice (Figure 3F). Four groups were tested: (a) saline control; (b) HD-SN-011 (4 mM) dissolved in saline; (c) free SN-011 dissolved in vehicle (10% DMSO, 40% PEG300, 5% Tween 80, and 45% saline); and (d) vehicle control. Mice injected with saline-dissolved HD-SN-011 exhibited normal scotopic A- and B-wave amplitudes comparable to those of saline controls, indicating preserved retinal function. In contrast, eyes injected with free SN-011 or its vehicle formulation showed markedly reduced ERG amplitudes, consistent with retinal toxicity induced by high DMSO concentrations and other organic cosolvents (Figure 3F and Supplemental Figure 2, A–D), indicating that high concentrations of DMSO and other organic cosolvents impair retinal function. In parallel, H&E staining and quantitative analysis of total retinal thickness performed 2 weeks after intravitreal injection revealed no significant differences among the 4 groups (Supplemental Figure 2E), indicating the absence of overt structural retinal damage under these conditions. Previous studies have also reported that intravitreal injection of DMSO-containing formulations can induce retinal ganglion cell apoptosis (38). Collectively, these findings demonstrate that HD-SN-011 exhibits excellent biocompatibility and substantially reduces the in vitro and in vivo cytotoxicity associated with free SN-011, supporting its potential as a safe and effective platform for intravitreal drug delivery.

### HD-SN-011 is rapidly and selectively taken up by MPs and suppresses cGAS/STING pathway activation in vitro.

**Figure 4:**
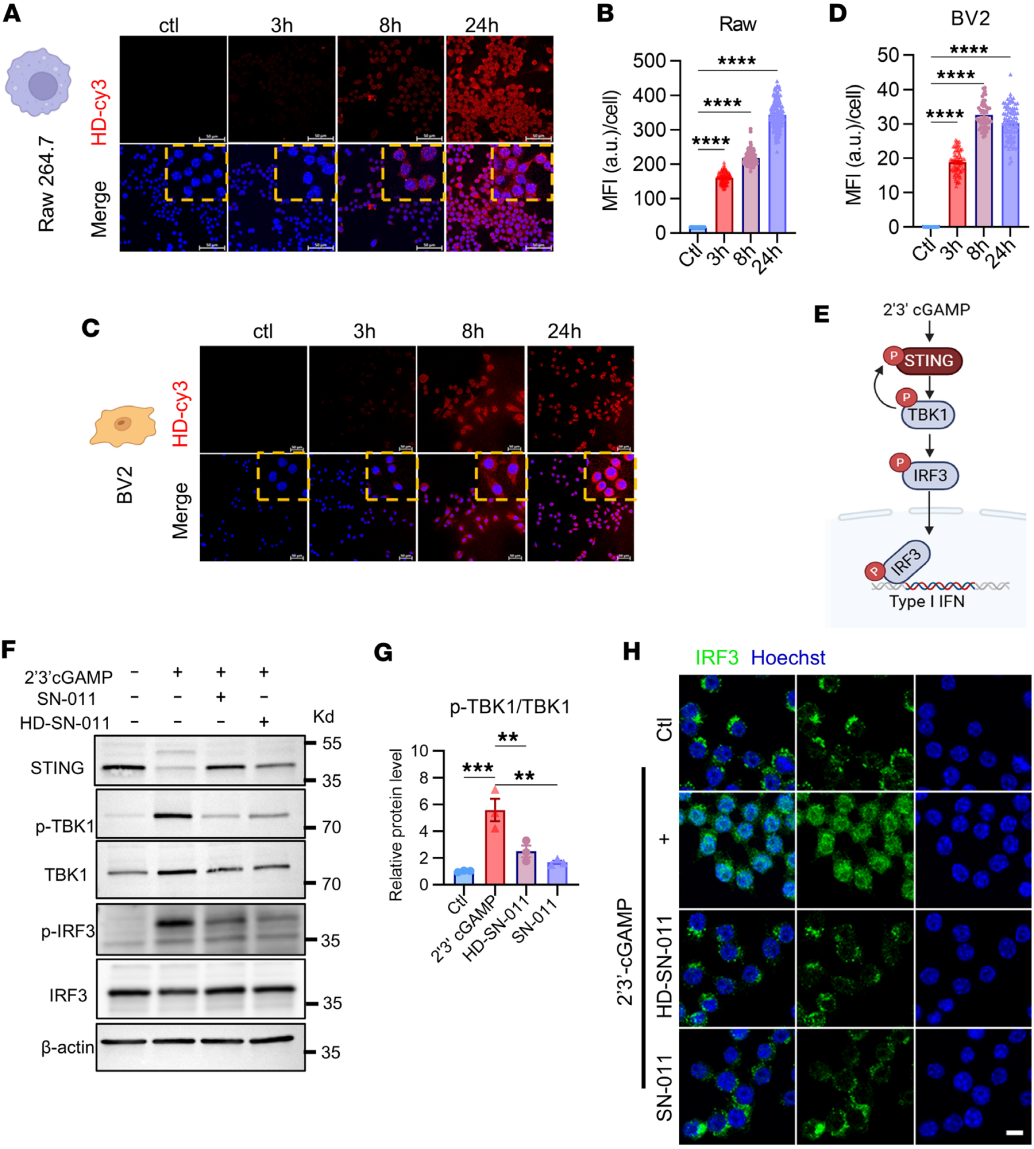
HD-Cy3 is efficiently taken up by microglia/macrophages and inhibits cGAS/STING activation in vitro. (**A** and **C**) Time-dependent uptake of HD-Cy3 in RAW264.7 macrophages (**A**) and BV2 microglia (**C**). Cells were incubated with HD-Cy3 (10 μg/mL) for 0, 3, 8, or 24 hours, then fixed and counterstained with Hoechst (blue). Representative images show a progressive increase in intracellular Cy3 signal (red). Yellow dashed boxes highlight regions of interest shown at higher magnification. Scale bars: 50 μm. (**B** and **D**) Quantification of mean fluorescence intensity (MFI) per cell demonstrates robust, time-dependent accumulation of HD-Cy3 in both RAW264.7 (**B**) and BV2 cells (**D**). (**E**) Schematic illustration of the cGAS/STING signaling cascade activated by 2′3′-cGAMP, leading to phosphorylation of TBK1 and IRF3 and induction of type I interferons. (**F**) Western blot analysis of STING pathway activation in RAW264.7 cells. Cells were pretreated with free SN-011 or HD-SN-011 (8 μg/mL) for 16 hours, followed by stimulation with 2′3′-cGAMP (25 nM) for 6 hours. β-Actin was used as a loading control. (**G**) Densitometric quantification of p-TBK1 normalized to total TBK1 in **F** (*n* = 3). (**H**) Immunofluorescent staining of IRF3 (green) in RAW264.7 cells. Cells were pretreated with free SN-011 or HD-SN-011 (8 μg/mL) for 16 hours, followed by stimulation with 2′3′-cGAMP (25 nM) for 6 hours. Nuclei were counterstained with Hoechst (blue). Scale bar: 10 μm. Statistical significance was assessed by 1-way ANOVA with Šídák’s multiple-comparison test. **P < 0.01; ***P < 0.001; ****P < 0.0001.

To evaluate whether HDs can be selectively and efficiently internalized by MPs, we synthesized a Cy3-labeled HD (HD-Cy3) (Supplemental Figure 3) and monitored its cellular uptake over time. Confocal imaging revealed minimal background fluorescence in untreated controls, whereas RAW264.7 macrophages and BV2 microglia exhibited clear intracellular red fluorescence as early as 3 hours after addition of HD-Cy3. The fluorescence intensity further increased at 8 and 24 hours, indicating efficient and sustained internalization of HD-Cy3 by phagocytes (Figure 4, A–D). We next examined the regulatory effects of HD-SN-011 on cGAS/STING pathway activation in cultured RAW264.7 macrophages. Stimulation of macrophages with 2′3′-cGAMP robustly activated the STING signaling cascade, as evidenced by increased TBK1 phosphorylation, elevated IRF3 phosphorylation, and promoted IRF3 nuclear translocation (Figure 4, E–H). Pretreatment with HD-SN-011 markedly attenuated these responses, substantially reducing p-TBK1 levels and inhibiting IRF3 nuclear accumulation (Figure 4, F–H). Notably, free SN-011 exhibited a stronger inhibitory effect at the same mass concentration (Figure 4, F and G), which is likely attributable to its immediately bioavailable form, whereas HD-SN-011 requires intracellular ester-bond hydrolysis for active-drug release. On the other hand, HD-SN-011’s dendrimer-based design enables efficient, targeted uptake by MPs and intracellular release of SN-011 over time, which would be expected to provide a longer duration of treatment effect. Collectively, these in vitro findings demonstrate that HDs are rapidly and effectively internalized by MPs, and that HD-SN-011 effectively suppresses activation of the cGAS/STING pathway, supporting its therapeutic potential.

### HD-Cy3 can be specifically taken up by reactive MPs in the laser-induced CNV model in vivo.

**Figure 5:**
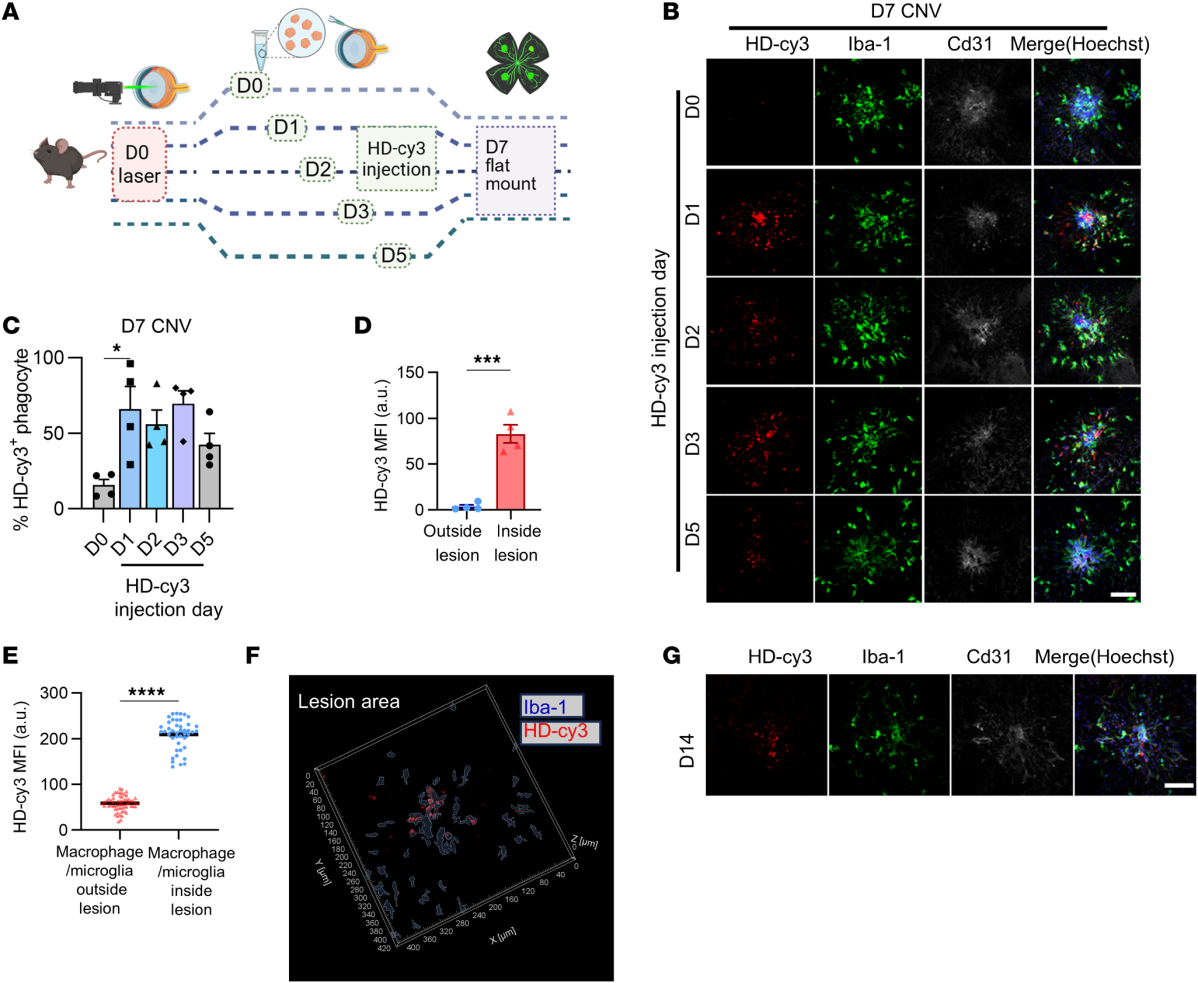
HD-Cy3 is specifically taken up by mononuclear phagocytes in laser-induced CNV in vivo. (**A**) Schematic of timeline of the experimental workflow. CNV was induced by laser photocoagulation on day 0 (D0). HD-Cy3 was intravitreally injected at different time points (D0, D1, D2, D3, or D5) after laser administration, and all eyes were collected on D7 after laser for flat-mount preparation and immunostaining. (**B**) Representative confocal images of CNV lesions from eyes injected with HD-Cy3 on D0, D1, D2, D3, or D5 and collected on D7. Flat-mounts were immunostained for IBA1 (green) and CD31 (gray), with nuclei counterstained with Hoechst (blue). Scale bar: 10 μm. (**C**) Quantification of the percentage of HD-Cy3^+^IBA1^+^ cells at D7 in eyes injected at the indicated time points after laser (*n* = 4 lesions). (**D**) Quantification of HD-Cy3 fluorescence intensity in **B**, comparing retinal quadrants inside and outside the CNV lesion (*n* = 4 lesions). (**E**) Fluorescence quantification of HD-Cy3^+^IBA1^+^ macrophages/microglia in **B**, comparing cells located inside the lesion (*n* = 44 IBA1^+^ cells) and outside the lesion (*n* = 60 IBA1^+^ cells). Cells were randomly selected from 4 lesion areas. (**F**) Three-dimensional reconstruction of the D7 CNV lesion demonstrating spatial colocalization of HD-Cy3 (red) with IBA1^+^ phagocytes (blue) within the neovascular core. (**G**) Representative confocal images showing HD-Cy3 uptake at D14 after HD-Cy3 injection on D1. Flat-mounts were immunostained for IBA1 (green) and CD31 (gray), with nuclei counterstained with Hoechst (blue). Scale bar: 10 μm. Data are represented as mean ± SEM. Statistical significance was assessed by 1-way ANOVA with Šídák’s multiple-comparison test (**C**) or 2-sided, unpaired *t* test (**D** and **E**). *P < 0.05; ***P < 0.001; ****P < 0.0001.

To determine whether PAMAM HDs can selectively target reactive phagocytes in vivo, we took advantage of the well-defined temporal dynamics of inflammatory cell recruitment in the laser-induced CNV model; MPs begin to infiltrate the lesion at around day 1 after laser injury and reach a peak at approximately day 3. For dendrimer localization studies, HD-Cy3 was intravitreally injected at different time points after laser induction (immediately, day 1, day 2, day 3, or day 5), and all eyes were collected on day 7 for parallel analysis (Figure 5A). Because unengulfed dendrimers are rapidly cleared via renal filtration within approximately 24 hours (28), HD-Cy3 will be present in the retina on day 7 after laser only in cells that have internalized them. Confocal imaging revealed that when HD-Cy3 was injected immediately after laser injury, virtually no signal was detected in the CNV region on day 7, indicating rapid clearance in the absence of reactive MPs and further supporting the selectivity and ocular safety of the HD platform (Figure 5, B and C). In contrast, injections performed on day 1, day 2, day 3, or day 5 resulted in pronounced accumulation of HD-Cy3 within IBA1^+^ MPs inside the CNV lesion; quantitative analysis showed that more than 70% of IBA1^+^ cells were HD-Cy3^+^ across all 4 groups (Figure 5, B and C). Three-dimensional reconstruction further confirmed tight spatial colocalization between HD-Cy3 and IBA1^+^ microglia/macrophages (Figure 5F). To further assess the spatial precision of HD localization, we compared HD-Cy3 distribution inside versus outside the CNV lesion. More than 80% of the total HD-Cy3 signal was confined to the CNV core (Figure 5D), indicating lesion-specific enrichment. Individual cell analysis of the fluorescence signal additionally revealed that IBA1^+^ cells located within the lesion exhibited approximately 3- to 4-fold higher HD-Cy3 fluorescence intensity compared with IBA1^+^ cells outside the lesion (Figure 5E), demonstrating that HD uptake is not a universal property of all retinal phagocytes but is tightly associated with the lesion-recruited reactive population. In addition, we examined the colocalization of HD-Cy3 with RPE cells after intravitreal injection on day 1 or day 4 following laser injury. Limited colocalization of HD-Cy3 signal with RPE cells was observed, indicating that HD-Cy3 is also taken up by RPE cells (Supplemental Figure 4), although to a lesser extent compared with MPs. Finally, to evaluate long-term intracellular retention, we injected HD-Cy3 on day 1 after laser induction and examined CNV lesions on day 14. HD-Cy3 signal remained detectable in IBA1^+^ cells surrounding the regressed lesions, indicating stable and prolonged intracellular persistence of the conjugate (Figure 5G). In summary, HDs target and are preferentially taken up by reactive phagocytes in CNV lesions in vivo.

### HD-SN-011 alleviates pathological neovascularization and vascular leakage in the laser-induced CNV model in vivo.

**Figure 6:**
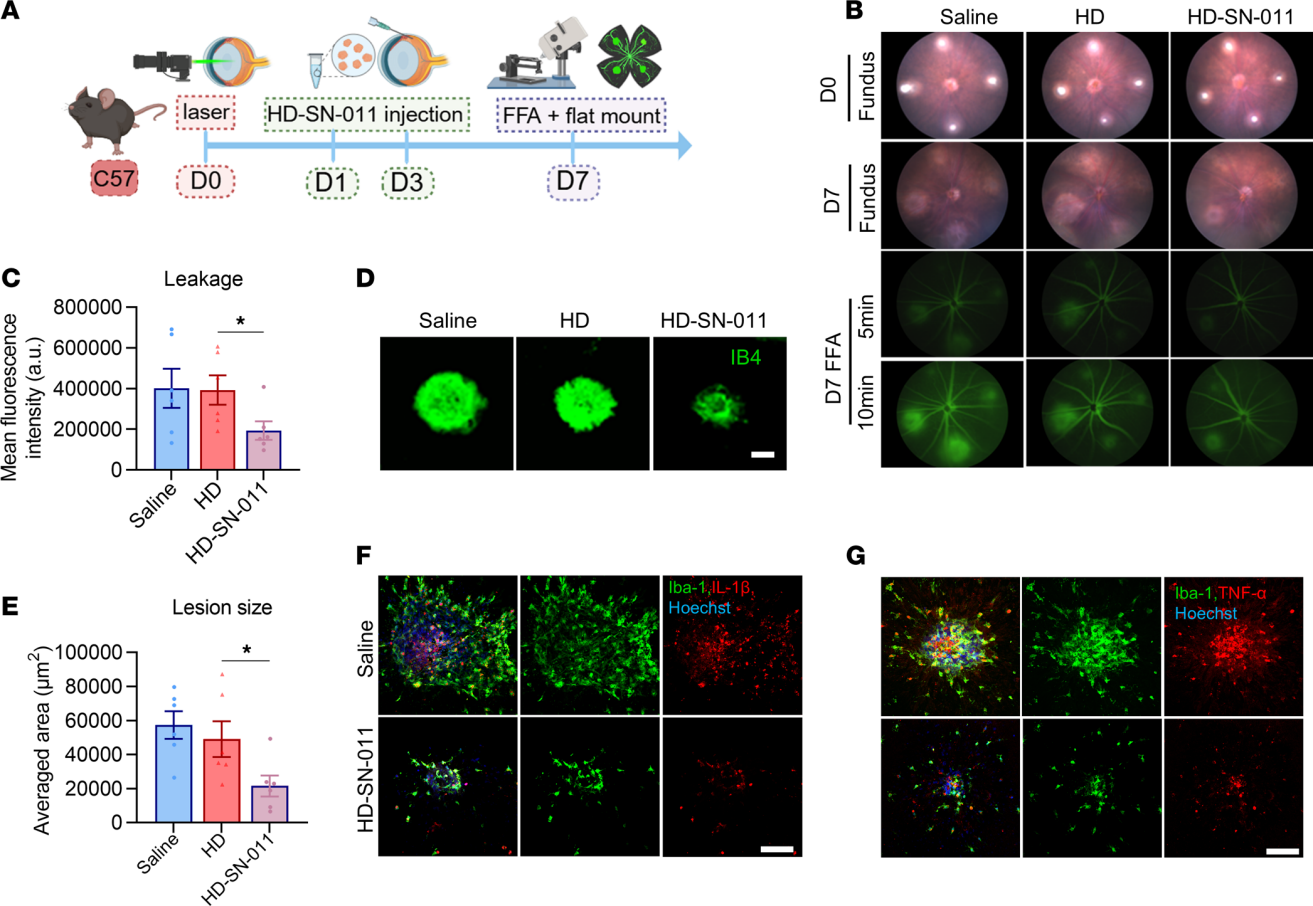
HD-SN-011 reduces CNV leakage, lesion size, and inflammatory signaling in the laser-induced CNV model. (**A**) Schematic timeline of the experiment. CNV was induced by laser photocoagulation on day 0 (D0), followed by intravitreal injection of saline or HD-SN-011 (4 mM, 1.85 mg/mL) on D1 or D3. On D7, fundus fluorescein angiography (FFA) and choroidal flat-mount analyses were performed. (**B**) Representative D0 fundus images after laser photocoagulation, D7 fundus images, and D7 FFA images acquired 5 and 10 minutes after intraperitoneal injection of sodium fluorescein in saline-, HD-, and HD-SN-011–treated mice. (**C**) Quantification of fluorescein leakage based on FFA images in **B**, calculated as the difference between fluorescence signal intensity at 10 minutes compared with 5 minutes after fluorescein injection (*n* = 7 mice). (**D**) Representative choroidal flat-mount images stained with IB4 lectin on D7 in saline- HD-, and HD-SN-011–treated eyes. Scale bar: 100 μm. (**E**) Quantification of CNV lesion area from choroidal flat-mounts in **D** (*n* = 7 mice). (**F** and **G**) Immunofluorescent staining of CNV lesions on D7. Flat-mounts were stained for IBA1 (green) together with IL-1β (**F**, red) or TNF-α (**G**, red), with nuclei counterstained using Hoechst (blue). Scale bars: 100 μm. Data are shown as mean ± SEM. Statistical analysis was performed using a 2-sided, unpaired *t* test. *P < 0.05.

To evaluate the therapeutic efficacy of HD-SN-011 in vivo, intravitreal injections of HD-SN-011 were performed 1 day and 3 days after laser induction, and the pathological features of CNV were analyzed on day 7 (Figure 6A). Fundus fluorescein angiography (FFA) showed no significant difference in vascular leakage between the saline and HD-only groups, whereas HD-SN-011 treatment markedly reduced vascular leakage. Quantitative analysis, calculated as the fluorescence intensity at 10 minutes minus that at 5 minutes, further confirmed that CNV leakage was reduced by approximately 50% following HD-SN-011 treatment (Figure 6, B and C). In addition, RPE-choroid flat-mount analysis demonstrated that HD-SN-011 treatment reduced CNV lesion size by approximately 60% compared with saline (55% compared with HD-only), indicating that HD-SN-011 effectively inhibits pathological neovascularization in vivo (Figure 6, D and E). Immunofluorescent staining showed strong IL-1β and TNF-α expression in IBA1^+^ MPs within CNV lesions in the saline group, whereas HD-SN-011 treatment markedly reduced the expression of both cytokines (Figure 6, F and G). Together, these findings demonstrate that HD-SN-011 significantly reduces vascular leakage and CNV lesion size in the laser-induced model, highlighting its potential as a therapeutic approach for nAMD through inhibition of both pathologic angiogenesis and CNV leakage.

### HD-SN-011 attenuates MP cGAS/STING activation and downstream type I interferon–mediated inflammation.

**Figure 7:**
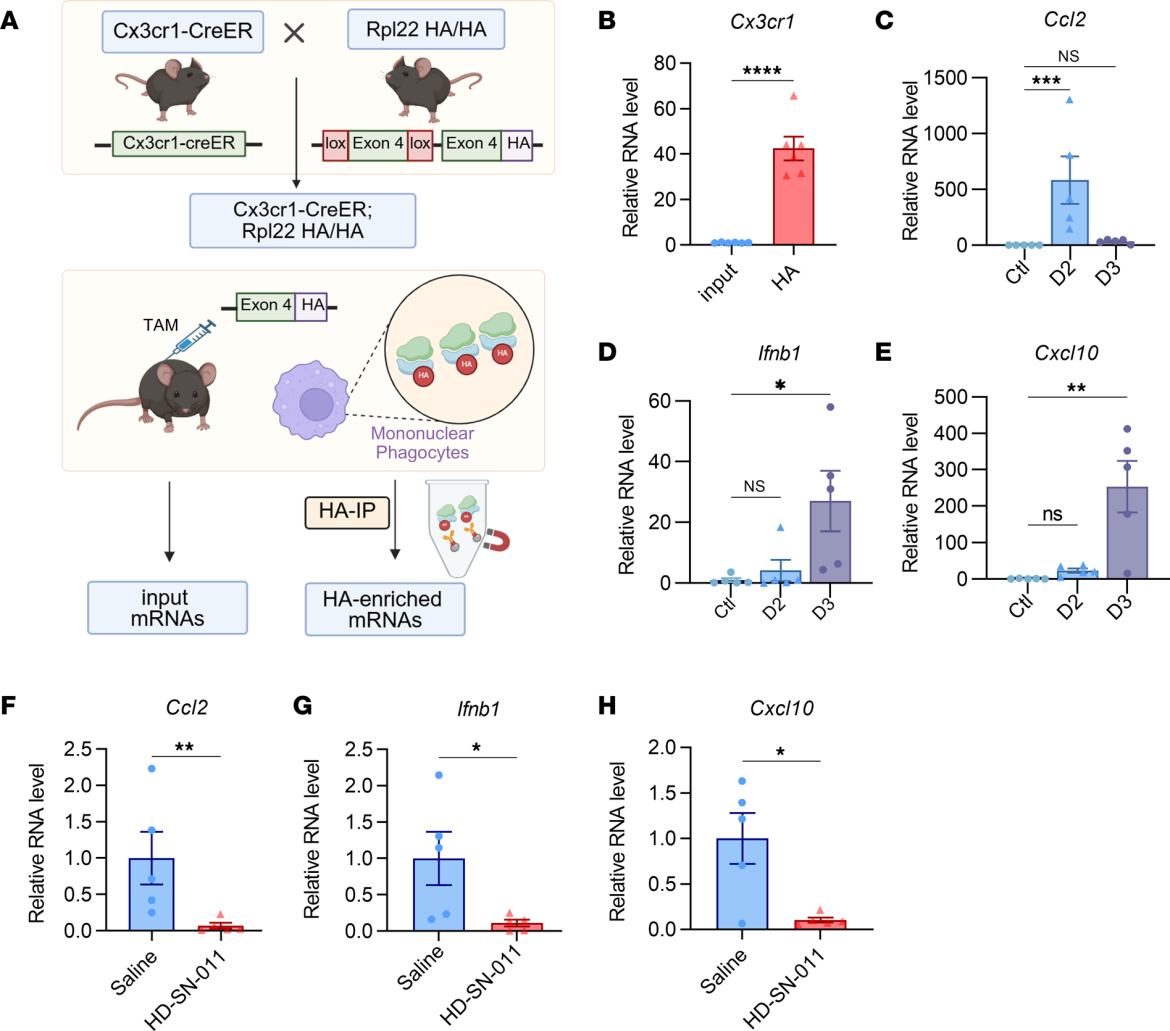
HD-SN-011 attenuates MP cGAS/STING activation and downstream type I interferon–related inflammatory gene expression. (**A**) Schematic of the Cx3cr1-CreER; Rpl22^HA/HA^ mouse model and HA-RiboTag enrichment workflow. Cx3cr1-CreER mice were crossed with Rpl22^HA/HA^ mice. Tamoxifen was administered to induce HA-tagged ribosome expression in Cx3cr1^+^ MPs. Retina-RPE-choroid complexes were collected to obtain input RNA and HA-immunoprecipitated (HA-IP) MP–enriched mRNAs. (**B**) qPCR validation of MP enrichment by comparing *Cx3cr1* expression between input and HA-IP RNA fractions. (**C**–**E**) qPCR analysis of *Ccl2* (**C**), *Ifnb1* (**D**), and *Cxcl10* (**E**) in HA-IP–enriched mRNAs at baseline (Ctl) and on day 2 (D2) or D3 after laser-induced CNV in saline-treated eyes. (**F**–**H**) Mice subjected to laser-induced CNV received intravitreal injection of saline or HD-SN-011 on D1, followed by qPCR analysis of *Ccl2* on D2 (**F**), and *Ifnb1* and *Cxcl10* on D3. (**G** and **H**). The saline control shown in **F** corresponds to the same D2 control in **C**, whereas the saline controls shown in **G** and **H** correspond to the same D3 controls as in **D** and **E**, respectively. Data are represented as mean ± SEM. Statistical significance was assessed by 1-way ANOVA with Šídák’s multiple-comparison test (**C**–**E**) or 2-sided, unpaired *t* test (**F**–**H**). NS, not significant. *P < 0.05; **P < 0.01; ***P < 0.001; ****P < 0.0001.

To confirm whether HD-SN-011 involves direct modulation of cGAS/STING signaling in MPs, we employed the Cx3cr1-CreER; Rpl22^HA/HA^ (RiboTag) mouse model (39), which enables selective isolation of ribosome-bound mRNA from MPs following tamoxifen-induced Cre activation (40, 41) (Figure 7A and Supplemental Figure 5A). HD-SN-011 or saline was intravitreally injected on day 1 after laser induction, and MP-specific translatome profiling was performed on days 2 and 3 after laser. Successful enrichment of MP-restricted transcripts such as *Cx3cr1* confirmed the effectiveness of the isolation strategy (Figure 7B). Quantitative PCR (qPCR) analysis revealed a time-dependent inflammatory response in saline-treated CNV eyes, in which *Ccl2* and *Tnf* were markedly upregulated on day 2, whereas *Ifnb1* and *Cxcl10* were significantly induced on day 3. HD-SN-011 treatment significantly suppressed the induction of *Ccl2* and *Tnf* on day 2 and markedly reduced *Ifnb1* and *Cxcl10* expression on day 3 (Figure 7, C–H, and Supplemental Figure 5, B and C). Collectively, these results demonstrate that HD-SN-011 suppresses cGAS/STING-associated inflammatory signaling and proinflammatory cytokine production in retinal MPs, thereby attenuating local inflammatory activation and contributing to its protective effect against pathological angiogenesis in AMD.

## Discussion

nAMD is a complex neurovascular disease driven by chronic inflammation and aberrant activation of the innate immune system within the retina and choroid (4). There has been an increasing appreciation for the important role played by both macrophages and microglia in the pathogenesis of nAMD and CNV (4). Evidence supporting the involvement of MPs in CNV has been grounded especially in experiments by multiple investigators involving depletion of macrophages, microglia, or both. Although such studies establish the pathogenic importance of MPs, treatments that target these cells will likely need to be based on pharmacologic modulation of these cells rather than depletion, given their important roles in retinal and systemic health (42–45). There have been extensive efforts to identify factors and pathways that regulate these cells, which can inform therapeutic efforts. The cGAS/STING signaling pathway has emerged for its critical regulatory role for the innate immune system, governing multiple cellular processes (16, 17). Importantly, this pathway is also active in non-immune cell types. Furthermore, active cGAS/STING signaling is beneficial in some contexts, including tumor surveillance, so that broad inhibition of this pathway could have adverse consequences (46). Fine-tuned targeting of cGAS/STING specifically in reactive MPs would therefore be ideal to maximize clinical translatability of cGAS/STING-based therapies. To address this need for selective targeting, we used a PAMAM HD for modulation of the cGAS/STING pathway in MPs in laser-induced CNV. Our group has demonstrated that these HDs target these cells at the site of pathology in many preclinical models in 6 species, including primates, using a biophysical mechanism that takes advantage of disease pathology (29–35). Our studies demonstrated selective delivery of HDs to reactive MPs in laser-induced CNV, direct modulation of MPs in this setting, and significant therapeutic beneficial effects on both CNV leakage and lesion size. Together, these results demonstrate that MP-specific cGAS/STING signaling is a primary pathogenic mechanism in CNV and provides proof of concept for specific targeting of MPs for the treatment of nAMD.

Initially identified as an essential pathway for immune defense against pathogens, the cGAS/STING pathway has received strong recognition for involvement in multiple disease contexts, including inflammatory conditions and antitumor immunity (16, 47). Although the pathogenic effects of cGAS/STING signaling have been characterized mostly in systemic and CNV conditions, this pathway has begun to receive attention for retinal diseases. In the oxygen-induced retinopathy (OIR) model (a model of ischemia-induced retinal angiogenic conditions such as proliferative diabetic retinopathy), pathologic preretinal neovascularization was reduced either in *Sting*^–/–^ mice or wild-type mice treated with a pharmacologic STING inhibitor (20, 48). Similarly, *Sting*^–/–^ mice exhibited reduced neovascular area in the laser-induced CNV model (20). In the OIR model, *Sting*^–/–^ mice had a significant reduction in the number of MPs, as measured by number of CD45^+^CD11b^+^ cells in the retina (48) or number of IBA1^+^ cells in the retinal neovascular region (20). Similarly, in the laser-CNV model, *Sting*^–/–^ mice had a significant reduction in the number of IBA1^+^ cells associated with the CNV lesion (20). This indicates that cGAS/STING is important in the regulation of angiogenesis in the eye and represents a promising therapeutic target. However, given the complex, multicellular nature of pathologic angiogenesis, there is a great need to identify and confirm the cellular context for pathogenic cGAS/STING signaling. Although inhibition of cGAS/STING is associated with reduced MP activation in these settings, it is certainly possible that this could be a secondary response.

For our interest in determining the specific importance of MP cGAS/STING, we used an approach for selective pharmacologic targeting of STING in these cells using PAMAM HDs with conjugation of the drug of interest to the surface hydroxyl groups (28). HD-conjugated drugs (and fluorescent tags) target reactive MPs in the retina and brain (29). In the current study in the setting of laser-induced CNV, using HD-Cy3, we found strongly selective targeting to the CNV lesion, with more than 80% of the signal within compared with outside the lesion. Furthermore, there was 3- to 4-fold higher HD-Cy3 fluorescence in IBA1^+^ cells within the CNV lesion, compared with outside. While HD preferentially accumulated in lesion-associated MPs, uptake by RPE cells was also observed, although to a lesser extent, in the laser-induced CNV model. This suggests that, although MPs are likely the predominant cellular target of HD-SN-011 in this setting, a potential contribution from RPE cannot be fully excluded. This is reminiscent of HD localization in the OIR model, in which HD-Cy5 demonstrated robust and selective localization in the MPs in the ischemic and neovascular retinal zones, but strikingly no localization with MPs in the perfused peripheral retina (30). The robust and selective localization of HD-conjugated molecules to MPs, particularly in the CNV disease area, provides the means for assessing the specific role of MP cGAS/STING using HD-SN-011, given the efficient and specific internalization of this drug by activated MPs. RiboTag-based translatome profiling confirmed that HD-SN-011 effectively suppressed activation of the cGAS/STING signaling pathway in retinal MPs, leading to downregulation of its downstream inflammatory mediators. Importantly, selective targeting of MP cGAS/STING by HD-SN-011 had beneficial effects in the laser-induced CNV mouse model, the most widely used experimental model of CNV (49). Treatment with HD-SN-011 significantly reduced both CNV lesion size (by approximately 60% compared with saline and 55% compared with HD-only) and CNV leakage (by ~50%), 2 clinically important endpoints in nAMD.

As a therapeutic strategy for nAMD, the HD approach for drug delivery provides multiple attractive advantages. Most important is the ability to robustly and preferentially target MPs (especially reactive MPs in areas of disease such as CNV), and avoid other cell types and thereby minimizing potential adverse effects. HD-conjugated drugs are quickly eliminated from the body, within 24–48 hours of drug administration, even in humans (32). With respect to inhibition of cGAS/STING, this selectivity for MPs is extremely important, as selective targeting is acknowledged to be highly desirable for clinical translation, in order to preserve beneficial effects of this pathway involving tumor surveillance and response to infection (19, 22–24). Notably, STING agonism is being pursued as a pharmacologic strategy to promote antitumor immunity (50–52). Beyond these considerations of cell-specific targeting, dendrimer conjugation provides important practical advantages in enhancing drug solubility and biocompatibility, which has high relevance for small-molecule STING inhibitors, such as C-176, H-151, and SN-011, which have poor aqueous solubility and high hydrophobicity. Consistent with other reports, our studies indicated that dissolving free SN-011 required high concentrations of DMSO, PEG300, and Tween 80, cosolvents that exhibit pronounced toxicity to the retina and other tissues. Our ERG analysis further confirmed that HD-SN-011 did not impair retinal electrophysiological function, indicating excellent ocular biocompatibility and safety. Collectively, these findings highlight HD conjugation as a powerful platform for modulating innate immune signaling within the retina and provide a strategy for localized immunotherapy in AMD and potentially other disease conditions.

## Methods

### Sex as a biological variable

Sex was not considered as a biological variable in the experimental design, data collection, or statistical analysis.

### Animals

Male and female mice under 9 weeks of age were used in this study. C57BL/6J, Rpl22^HA/HA^ [B6J.129(Cg)-Rpl22^tm1.1Psam^/SjJ; catalog 029977], and CX3CR1-CreER (catalog 020940) mice were purchased from the Jackson Laboratory. Mice were housed under controlled conditions (20°C–23°C, 12-hour light/dark cycle).

### Reagents, resources, and antibodies

The reagents, resources, and antibodies are listed in Supplemental Table 1.

### Laser-induced CNV model

The mouse laser-induced CNV model was established as previously described (49), with minor modifications. Mice were anesthetized with a cocktail of ketamine, xylazine, and acepromazine dissolved in PBS, followed by pupillary dilation with 1% tropicamide. To maintain corneal clarity and prevent drying, a drop of lubricating eye solution (NDC 77790-022-15, VISTA Advanced) was applied to the ocular surface. Laser photocoagulation was performed using the Phoenix Micron III retinal imaging system. The following laser parameters were used: spot size 50 μm, duration 70 ms, and power 240 mW. Successful rupture of Bruch’s membrane was confirmed by the immediate appearance of a cavitation bubble. Laser burns were applied approximately 2 optic disc diameters away from the optic nerve head while avoiding major retinal vessels. Lesions with subretinal or vitreous hemorrhage, or without cavitation bubble formation, were excluded from analysis. For therapeutic efficacy studies, C57BL/6J mice received 4 laser spots per eye and were assigned to independent experimental groups. Intravitreal injections (1 μL/eye) were administered on days 1 and 3 after laser. On day 7, vascular leakage was assessed by FFA after intraperitoneal injection of 1 mL of 5% sodium fluorescein, with one eye randomly selected from each mouse for imaging at 5 and 10 minutes. Leakage was quantified using ImageJ/Fiji (NIH) as the fluorescence intensity at 10 minutes minus that at 5 minutes. Mice were then euthanized, and the RPE-choroid complex was collected, stained with IB4 lectin–Alexa Fluor 488 (Thermo Fisher Scientific), flat-mounted, and imaged. CNV lesion area was quantified from valid laser spots using ImageJ/Fiji. For each mouse, one data point represents the average of 4 lesions per eye, excluding invalid lesions due to failed burns, hemorrhage, retinal detachment, or unclear lesion borders. For RiboTag mice used in MP translatome profiling, 20 laser spots were applied per eye. After lasering, mice were placed on a heating pad and monitored until full recovery from anesthesia.

### Single-cell RNA-seq analysis

Single-cell RNA-seq data for human retinal and choroidal cell populations were obtained through the Spectacle platform, an interactive resource for ocular single-cell transcriptomic analysis (53). The specific dataset used in this study was derived from Voigt et al. (54). Expression levels of cGAS across annotated cell populations were extracted from this dataset and visualized within the Spectacle platform.

### Immunofluorescent staining

For eye tissue staining, eyeballs were enucleated and fixed in 4% paraformaldehyde for 50 minutes, followed by a sucrose gradient dehydration at 4°C: 10% sucrose for 2 hours, 20% sucrose for 2 hours, and 30% sucrose overnight. The eyeballs were then embedded in optimal cutting temperature (OCT) compound and stored at –80°C. Tissues were cryosectioned at a thickness of 12 μm, blocked with 5% BSA in PBST (0.1% Tween 20) for 1 hour at room temperature, and incubated with primary antibodies overnight at 4°C. After washing with TBS containing 0.1% Tween 20, sections were incubated with Alexa Fluor–conjugated secondary antibodies (Thermo Fisher Scientific) for 1 hour at room temperature in the dark. Hoechst (Thermo Fisher Scientific) was used for nuclear staining.

For cell staining, cultured cells were fixed with 4% paraformaldehyde at 37°C for 15 minutes and washed 3 times with PBS. Cells were permeabilized with 0.25% Triton X-100 in PBS for 10 minutes and blocked with 3% BSA in PBST for 1 hour at room temperature. Primary antibodies diluted in 3% BSA in PBST were incubated overnight at 4°C, followed by incubation with Alexa Fluor–conjugated secondary antibodies for 1 hour at room temperature in the dark. Images were captured using a Zeiss LSM 710 laser-scanning confocal microscope.

### Western blot analysis

For Western blot analysis, cells or the RPE-choroid complex from mouse retina were lysed in RIPA buffer (R0278, Sigma-Aldrich) supplemented with a protease inhibitor cocktail (P8340, Sigma-Aldrich) and a phosphatase inhibitor cocktail (HY-K0021, MCE). The lysates were further disrupted by sonication and centrifuged to remove debris, after which protein concentration was quantified using a DC Protein Assay Kit (Bio-Rad). Equal amounts of protein (25 μg) were resolved on TGX precast gels (5671083, Bio-Rad) and transferred onto nitrocellulose membranes. Membranes were incubated with the indicated primary antibodies overnight at 4°C, followed by incubation with HRP-conjugated secondary antibodies. Immunoreactive bands were detected using a chemiluminescence detection kit (Thermo Fisher Scientific).

### NMR

NMR spectra were recorded on a Bruker 500 MHz spectrometer. Proton chemical shifts (δ) are reported in ppm and were referenced to the residual protic solvent peak of DMSO-*d_6_* at 2.50 ppm.

### HPLC

HPLC (Waters Corporation) is equipped with a 717 Plus autosampler and a 2998 photo-diode array detector, interfaced with Waters Empower software, and the column used is a symmetry 300 C18 (4.6 mm × 250 mm). For PAMAM-G4-OH, starting conjugates were monitored at 210 nm for dendrimer detection. For the drug SN-011 and the dendrimer conjugate (HD-SN-011), 260 nm was used for purity tests. For analytical detection, solvent A (water/0.1% TFA) and solvent B (acetonitrile/0.1% TFA) were used. A gradient flow was used starting with 100% A (water) for 5 minutes and to 75% B (acetonitrile) at 25 minutes, 100% B (acetonitrile) at 30 minutes, returning to 10% A (water) with 1 mL/min as the flow rate. Detection at 210 nm or 260 nm was used to report the purity of the analyte.

### DLS and ζ potential measurements

The particle size and ζ potential of HD-SN-011 were determined by using a Zetasizer Nano ZS (Malvern Instrument Ltd.). The HD concentrations for DLS measurement were 0.1 mg/mL in deionized water (UV-transparent disposable cuvette). Samples dissolved in water were sonicated and filtered through 0.2 μm syringe filters (PES membrane) before analysis. Measurement type: manual. Number of measurements: 3. Number of runs per measurement was 16.

For ζ potential measurement, the solvent condition was 10 mM NaCl solution, which was filtered through 0.2 μm syringe filters (PES membrane) before analysis. Number of measurements: 3. Number of runs per measurement: 20.

### Synthesis of HD-SN-011

The synthesis of HD-SN-011 was carried out in 4 consecutive steps while retaining all intermediate purification steps.

#### Synthesis of HD-GABA-Boc.

Hydroxyl-terminated PAMAM-G4 HD, supplied as an approximately 16% w/v methanol solution, was first evaporated and freeze-dried, after which 1 g of HD (0.07 mmol) was dissolved in 10 mL of anhydrous dimethyl formamide (DMF) and reacted with Boc-GABA-OH (113.81 mg, 0.56 mmol) that had been preactivated with EDC·HCl (161.03 mg, 0.84 mmol) and 4-dimethylaminopyridine (DMAP; 102.63 mg, 0.84 mmol). The reaction mixture was stirred for 36 hours at room temperature, dialyzed against DMF using a 1 kDa MWCO membrane for 24 hours, followed by extensive dialysis against water, and then lyophilized to afford the protected intermediate (compound 2, HD-GABA-Boc) as a white powder. ^1^H-NMR (500 MHz, DMSO-*d_6_*) δ 8.32–7.78 (m, internal amide H), 6.8 (s, GABA amide H) 4.70 (s, dendrimer surface-OH) 3.99 (d, ester linked-CH_2_), 3.39 (d, dendrimer-CH_2_), 3.2–3 (m, dendrimer-CH_2_), 2.94 (m, dendrimer-CH_2_), 2.7–2.5 (m, dendrimer-CH_2_), 2.43–2.40 (m, dendrimer-CH_2_), 2.1–2.24 (m, dendrimer-CH_2_), 1.64–1.57 (m, GABA-linker-CH_2_), 1.34 (s, -Boc protons).

#### Synthesis of HD-GABA-NH_2_.

The -Boc deprotection to obtain compound 3 (HD-GABA-NH_2_) was achieved by dissolving compound 2 (200 mg) in 5 mL anhydrous DCM, cooling the solution on ice, and slowly adding 25% TFA in DCM under vigorous stirring. The reaction was stirred overnight, followed by the addition of anhydrous methanol, evaporation under reduced pressure, and co-evaporation with toluene to fully remove residual TFA. ^1^H-NMR (500 MHz, DMSO-*d_6_*) δ 8.40–7.8 (m, internal amide H), 4.67 (s, dendrimer surface-OH), 4.1–3.9 (m, ester linked-CH_2_), 3.4–3.2 (m, dendrimer-CH_2_), 3.3–3.1 (m, dendrimer-CH_2_), 2.97–2.73 (m, dendrimer-CH_2_), 2.73–2.55 (m, dendrimer, -CH_2_), 2.43–2.40 (m, dendrimer-CH_2_), 2.1–2.24 (m, dendrimer-CH_2_), 1.8–1.7 (m, GABA-linker-CH_2_).

#### Synthesis of SN-011–succinic acid conjugate.

SN-011 (40 mg, 0.09 mmol) was dissolved in anhydrous DMF and reacted with 1.5 mol excess succinic anhydride (13.51 mg, 0.14 mmol) and 1 mol excess triethylamine for 24 hours at room temperature, the product (compound 5) was isolated by phase separation between DCM and water, washed twice with 1N HCl, saturated brine, dried over anhydrous sodium sulfate, and concentrated under reduced pressure to obtain the succinic acid–modified SN-011 intermediate. The product was then used without further purification.

#### Synthesis of HD-SN-011.

Compound 3 was dissolved in anhydrous DMF and reacted with compound 5 (7 mol excess), which had been preactivated with EDC·HCl and NHS in DMF. After completion, the reaction mixture was purified by dialysis (1 kDa MWCO) first against DMF and then extensively against water, followed by freeze-drying to yield the final conjugate HD-SN-011 as white flakes. ^1^H-NMR (500 MHz, DMSO-*d_6_*) δ 8.4–7.6 (m, internal amide H), 7.4–6.3 (aromatic protons of SN-011), 4.70 (s-broad, dendrimer surface-OH), 3.99–3.9 (m, ester linked-CH_2_), 3.42–3.3 (d, dendrimer-CH_2_), 3.4-3.1 (m, dendrimer-CH_2_), 2.94–3.02 (m, dendrimer-CH_2_), 2.82–2.51 (m, dendrimer, -CH_2_), 2.45–2.3 (m, dendrimer-CH_2_), 2.0–2.25 (m, dendrimer-CH_2_), 1.6 (m, GABA-linker-CH_2_).

#### Synthesis of G4-OH-alkyne.

PAMAM-G4-OH, compound 1 (100 mg, 0.007 mmol), was partially functionalized with alkyne termini by reacting with 5-hexynoic acid (5 mg, 0.042 mmol) using EDC (8.5 mg, 0.042) and DMAP (5.13 mg, 0.042 mmol) as coupling agents in anhydrous DMF at room temperature for 48 hours, yielding the alkynated intermediate, compound 2. The product was purified by dialysis against DMF and water to remove excess reagents and by-products. Then the alkyne-functionalized dendrimer was conjugated with azide-modified Cy3 dye (Cy3-N_3_) via a copper(I)-catalyzed azide-alkyne cycloaddition (CuAAC) “click” reaction. The reaction was performed in a DMF/H_2_O/THF solvent mixture containing CuSO_4_·5H_2_O and sodium ascorbate as the catalytic system, under microwave irradiation at 50°C for 12 hours. The resulting HD-Cy3, compound 3, featured a stable triazole link between the dendrimer and the Cy3 chromophore. The conjugate was purified by extensive dialysis (MWCO 1 kDa) and lyophilized to obtain a pink solid. The conjugation of Cy3 to HD was confirmed by and ^1^H-NMR signals corresponding to the triazole and Cy3 aromatic protons. ^1^H-NMR (500 MHz, DMSO-*d_6_*) δ 8.42–7.78 (m, internal amide H), 7.60 (s, Cy3 H), 7.43 (s Cy3 H), 7.26 (s Cy3 H), 6.45 (s Cy3 H), 3.50–2.75 (m, dendrimer CH_2_), 1.64–1.59 (s, 31H), 1.84–0.86 (m, Cy3 H). HPLC C18 retention time (MeCN in H_2_O with 0.1% TFA, linear gradient, 35 minutes). HPLC C_18_ retention time: 3.0 minutes.

### Synthesis of HD-Cy3 (compound 3)

A solution of compound 4 (287 mg, 0.0048 mmol) in DMF (5 mL) was treated with DIPEA N,N-diisopropylethylamine (Sigma-Aldrich) to adjust pH of the reaction mixture (~7.0–7.5). Then, the reaction was treated with Cy3-NHS ester (8.7 mg, 0.0115 mmol,1.2 eq) and stirred at room temperature for 12 hours. It was then dialyzed against DMF for 12 hours followed by against water for 24 hours. The aqueous layer was frozen and lyophilized to yield the desired product 5 as a blue solid (yield 85%).

### In vitro drug release assay

The lysosome-simulated drug release was performed under in vitro conditions by simulating the pH as 5.5 with 0.5 M sodium citrate buffer and the ester bond–cleaved drug release is mediated by externally supplied porcine liver esterase. In a typical experiment, 1 mg/mL solution of HD-SN-011 dissolved in citrate buffer was added with 2 units of enzyme per mg of HD-SN-011 and incubated at 37°C. The samples were withdrawn at regular intervals, and the enzymes were quenched with equal volume of acetonitrile. The samples were then analyzed by HPLC at 260 nm for the drug release (SN-011).

### Cell culture and treatment

The mouse-derived macrophage cell line RAW 264.7, BV2 microglial cells, and mouse photoreceptor-derived 661W cells were obtained from ATCC and cultured in high-glucose Dulbecco’s modified Eagle medium (DMEM, Gibco) supplemented with 10% fetal bovine serum (FBS, Gibco) and 1% penicillin-streptomycin (Gibco). HUVECs were obtained from ATCC and cultured in endothelial cell growth medium (EGM-2, Lonza) supplemented with growth factors and 2% FBS according to the manufacturer’s instructions. All cells were maintained in a humidified incubator at 37°C with 5% CO_2_.

For drug treatment experiments, cells were seeded into 96-well plates and allowed to adhere overnight, followed by treatment with varying concentrations of SN-011 (dissolved in DMSO) or HD-SN-011 (dissolved in PBS) for 16 hours. The final concentration of DMSO in all groups was kept below 0.1%. Cell toxicity was evaluated using the CellTiter-Glo Luminescent Cell Viability Assay (Promega) following the manufacturer’s protocol.

### Ribosome immunoprecipitation (RiboTag)

For tamoxifen-inducible recombination, mice were administered tamoxifen before laser-induced CNV. Specifically, mice received tamoxifen (10 mg/mL, i.p.) 4 weeks before laser photocoagulation at 1 mg per day for the first 3 consecutive days, followed by 2 mg per day on days 4 and 5. In addition, 1 week before laser photocoagulation, mice received tamoxifen (10 mg/mL, i.p.) at 1 mg per day for 3 consecutive days. The RiboTag method was used to isolate polysome-bound mRNA from retinal tissues, following previously published protocols (41, 55). Briefly, the retina-RPE-choroid complex was homogenized on ice in 400 μL of polysome lysis buffer containing 50 mM Tris (pH 7.5), 100 mM KCl, 12 mM MgCl_2_, 1% NP-40, 1 mM DTT, 200 U/mL RNasin, 1 mg/mL heparin, 100 μg/mL cycloheximide, and a protease inhibitor cocktail. The lysates were centrifuged at 15,300*g* for 10 minutes at 4°C, and 10 μL of the supernatant was reserved as the input control. The remaining supernatant was incubated overnight at 4°C on an orbital shaker with 25 μL of anti-HA antibody–conjugated magnetic beads (M180-11, MBL International) to immunoprecipitate HA-tagged ribosome-bound complexes. The following day, the beads were washed 4 times with high-salt buffer (50 mM Tris pH 7.5, 300 mM KCl, 12 mM MgCl_2_, 1% NP-40, 1 mM DTT, 100 μg/mL cycloheximide), then resuspended in 350 μL of RLT buffer containing β-mercaptoethanol. After vortexing, the samples were placed on a magnetic stand and the supernatant was transferred to a new RNase-free tube. RNA was purified using the RNeasy Micro Kit (QIAGEN) according to the manufacturer’s instructions. Single-stranded cDNA was synthesized from the isolated RNA using M-MLV Reverse Transcriptase (Invitrogen), and qPCR was performed using SYBR Green PCR Master Mix (Invitrogen) on a StepOnePlus Real-Time PCR System (Applied Biosystems).

### ERG

Scotopic ERGs were recorded using a Celeris ERG system (Diagnosys) as previously described (56). Mice were dark adapted overnight (≥12 hours), and all procedures were performed under dim red light. Animals were anesthetized with an intraperitoneal injection of ketamine (100 mg/kg) and xylazine (10 mg/kg), followed by topical application of 1% tropicamide to induce mydriasis. A thin layer of 2.5% hypromellose was applied to the cornea immediately before placing the recording electrodes to maintain hydration and conductivity. Corneal light-guiding electrodes were positioned on both eyes, with a ground electrode inserted subdermally into the forehead between the eyes and a reference electrode inserted into the hip. Scotopic ERG responses were evoked using flash intensities of 0.0125, 0.250, 0.1, 1.000, and 10.000 cd·s/m^2^. For all flash intensities, 10 traces were recorded and averaged.

### Statistical analysis

GraphPad Prism 9 was used for data analysis. Unpaired *t* tests were performed between 2 groups. One-way or 2-way ANOVA was used for multiple group comparisons. Data are presented as mean ± SEM. A *P* value of less than 0.05 was considered significant. NS, not significant.

### Study approval

All animal experiments were approved by the Institutional Animal Care and Use Committee (IACUC) of the Johns Hopkins University School of Medicine and conducted in accordance with the Association for Research in Vision and Ophthalmology (ARVO) Statement for the Use of Animals in Ophthalmic and Vision Research.

### Data availability

All data are available in the main text or the supplemental materials. Values for all data points in graphs are reported in the Supporting Data Values file. Further information and requests for resources should be directed to and will be fulfilled by the lead contact, Elia Duh (eduh@jhmi.edu).

## Author contributions

EJD and RMK conceived and supervised the work. EJD and RMK designed research. LS, DC, HC, KR, LZ, YC, NK, ZX, and WL performed research. LS and DC analyzed data and generated the figures. LS, DC, RMK, and EJD wrote the manuscript and revised the manuscript.

## Conflict of interest

RMK is a co-inventor on over 190 patents (awarded and pending); many patents on dendrimer technologies, including US Patent US10369124, are licensed to the start-ups he co-founded, including Ashvattha Therapeutics, for retinal and other disorders. RMK, his wife, and EJD own stock in Ashvattha Therapeutics. This arrangement has been reviewed and approved by Johns Hopkins University in accordance with its conflict-of-interest policies.

## Funding support

This work is the result of NIH funding, in whole or in part, and is subject to the NIH Public Access Policy. Through acceptance of this federal funding, the NIH has been given a right to make the work publicly available in PubMed Central.

- NIH grants R01EY035897 and R01EY035549 (to EJD).
- NIH core grant P30EY001765 (Imaging and Microscopy Core Module to EJD).
- Altsheler-Durell Foundation (to EJD).
- Arnall Patz Distinguished Professorship (to RMK).
- RPB Unrestricted Grant (Wilmer Eye Institute, The Johns Hopkins University, to EJD and RMK).

## Supplementary Material

Supplemental data

Unedited blot and gel images

Supporting data values

## Version 1 08/10/2026

Electronic publication

## Version 2 08/11/2026

Missing author name elements restored in HTML version
