# Supplementary material for: Mononuclear phagocyte–specific cGAS/STING targeting suppresses experimental choroidal neovascularization
Source: JCI Insight. 2026 Aug 10;11(15):e203452. doi: 10.1172/jci.insight.203452 (PMC13464012; doi:10.1172/jci.insight.203452)
Supplement: Supplementary file 1 [file jciinsight-11-203452-s328.pdf]

Supplementary Fig.1

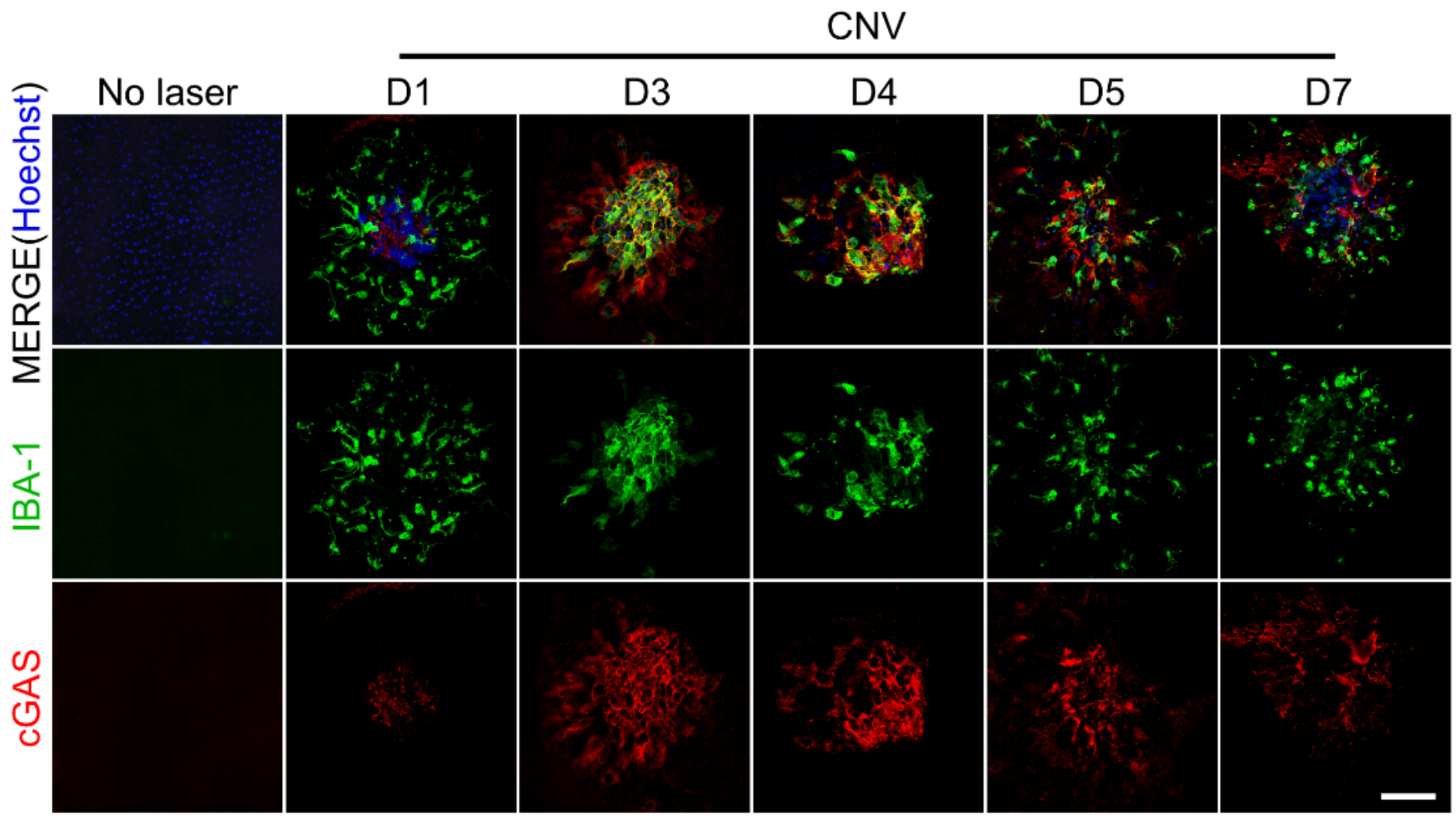

Supplementary Fig. 1. Temporal pattern of cGAS and IBA1 staining in RPE/choroid flat mounts after laser-induced CNV.

Representative immunofluorescence images of RPE/choroid flat mounts from no-laser control eyes and eyes collected at day 1, day 3, day 4, day 5, and day 7 after laser-induced CNV. Flat mounts were stained for IBA1 (green), cGAS (red), and Hoechst (blue) Scale bar, 100 µm.

Supplementary Fig.2

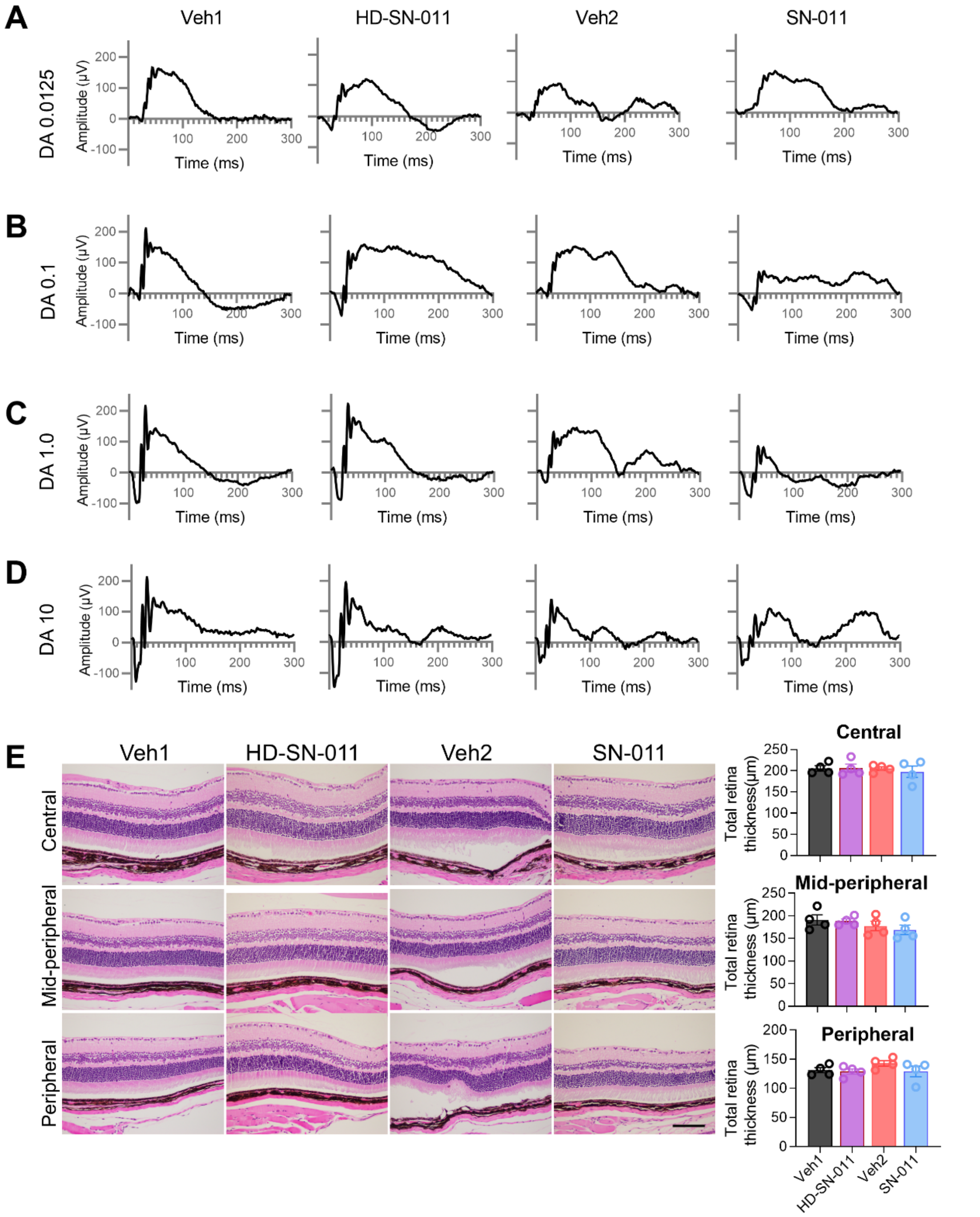

Supplementary Fig. 2. Ocular safety evaluation following intravitreal administration of HD-SN-011 or free SN-011.

(A-D) Mice received intravitreal injections of vehicle 1 (saline), HD-SN-011 (dissolved in vehicle 1), vehicle 2 (10% DMSO + 40% PEG300 + 5% Tween-80 + 45% saline), or free SN-011 (dissolved in vehicle 2). Dark-adapted ERG recordings were performed one week after laser induction under different flash intensities: DA10, DA1.0, DA0.1,

and DA0.0125. Time scale: 0–300 ms. **(E)** Representative H&E-stained retinal sections from central, mid-peripheral, and peripheral retina collected 2 weeks after intravitreal injection of Veh1, HD-SN-011, Veh2, or free SN-011, with corresponding quantification of retinal thickness in each region. Scale bar, 100 μm. Data are shown as mean ± SEM.

Supplementary Fig.3

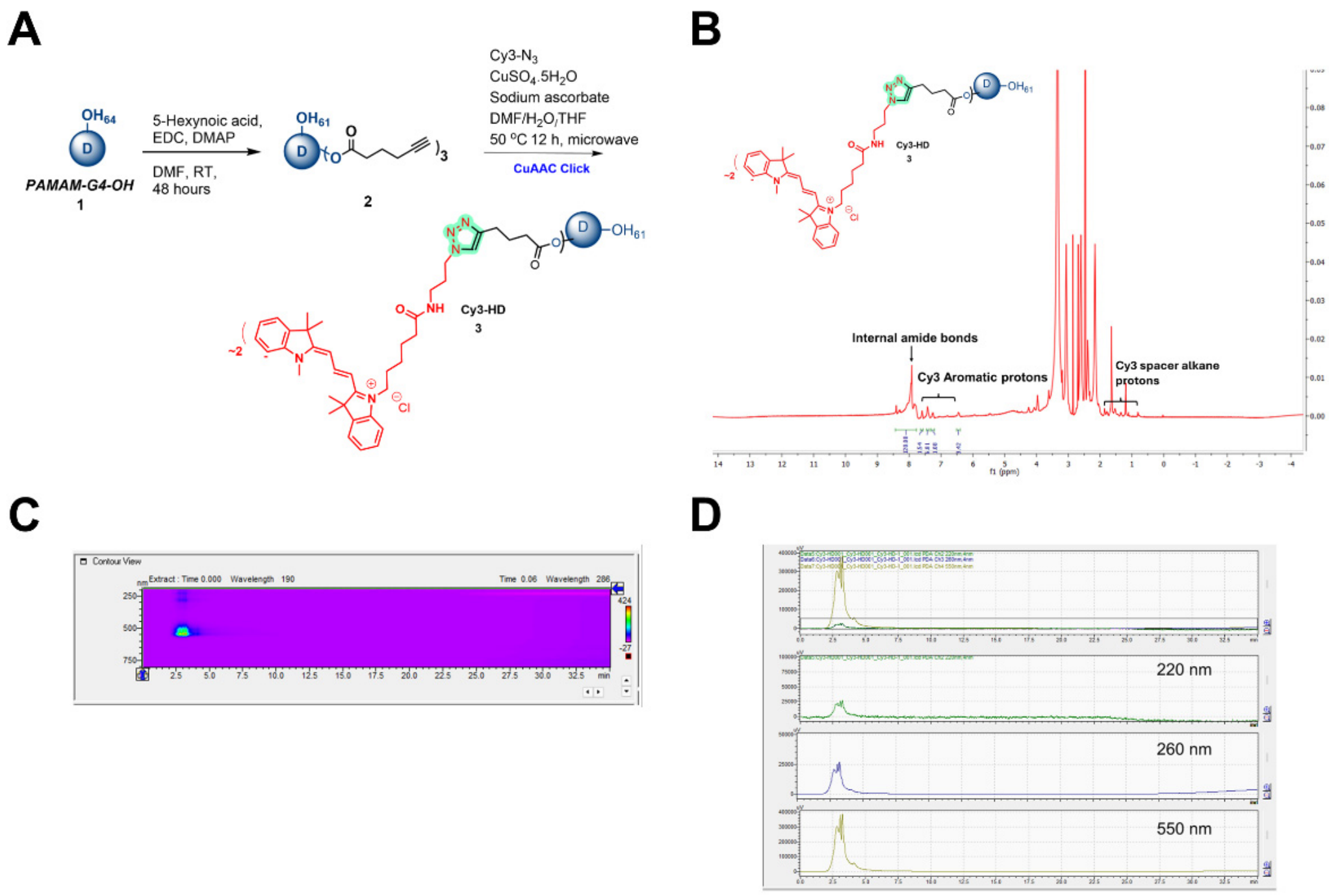

Supplementary Fig 3. Synthesis and Characterization of Cy3-Labeled Hydroxyl-Terminated PAMAM Dendrimer (Cy3-HD)

(A) Schematic-Synthesis of Cy3-labeled hydroxyl-terminated PAMAM dendrimer (Cy3-HD). Generation 4 hydroxyl-terminated PAMAM dendrimer (1) was partially functionalized with 5-hexynoic acid using EDC and DMAP coupling in DMF at room temperature for 48 h to yield alkyne-modified intermediate (2). The resulting alkyne-functional dendrimer was then conjugated with Cy3-azide (Cy3–N_3) via Cu(I)-catalyzed azide–alkyne cycloaddition (CuAAC click reaction) using $CuSO_4{\cdot}5H_2O$ and sodium ascorbate in a DMF/H_2O/THF mixture under microwave heating (50 °C, 12 h) to produce the Cy3-labeled dendrimer (3, Cy3-HD). The reaction introduces triazole linkages (green highlight) connecting the fluorescent dye (red) to the dendrimer. **(B)** 1H NMR spectrum of Cy3–HD in DMSO-d6 showing characteristic peaks corresponding to internal amide protons, aromatic protons of Cy3, and Cy3 alkyl spacer

protons, confirming successful conjugation of Cy3 to the dendrimer. **(C)** 2D contour HPLC chromatogram displaying a dominant single peak corresponding to Cy3–HD, indicating high purity and efficient conjugation. **(D)** HPLC chromatograms of Cy3–HD monitored at 220 nm, 260 nm, and 550 nm demonstrate co-elution of Cy3 absorbance with dendrimer-associated peaks, confirming covalent attachment of the Cy3 fluorophore to the hydroxyl dendrimer backbone.

Supplementary Fig.4

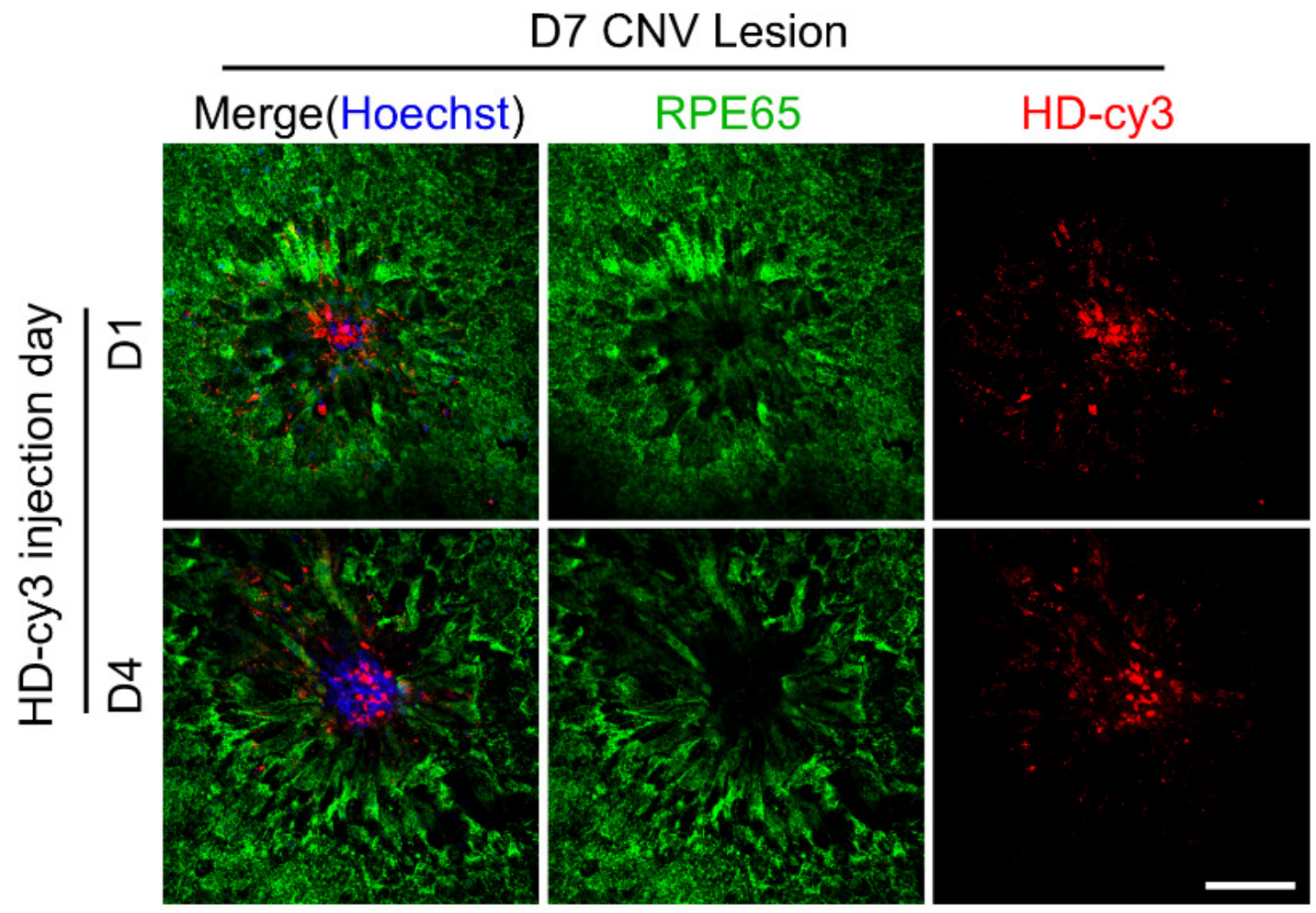

Supplementary Fig. 4. Limited colocalization of HD-Cy3 with RPE cells in laser-induced CNV lesions.

Representative confocal images of CNV lesions collected at day 7 after laser injury. Mice received intravitreal injection of HD-Cy3 on D1 or D4 after laser photocoagulation. RPE cells were labeled with RPE65 (green), HD-Cy3 is shown in red, and nuclei were stained with Hoechst (blue). Scale bar, 100 μm.

Supplementary Fig. 5

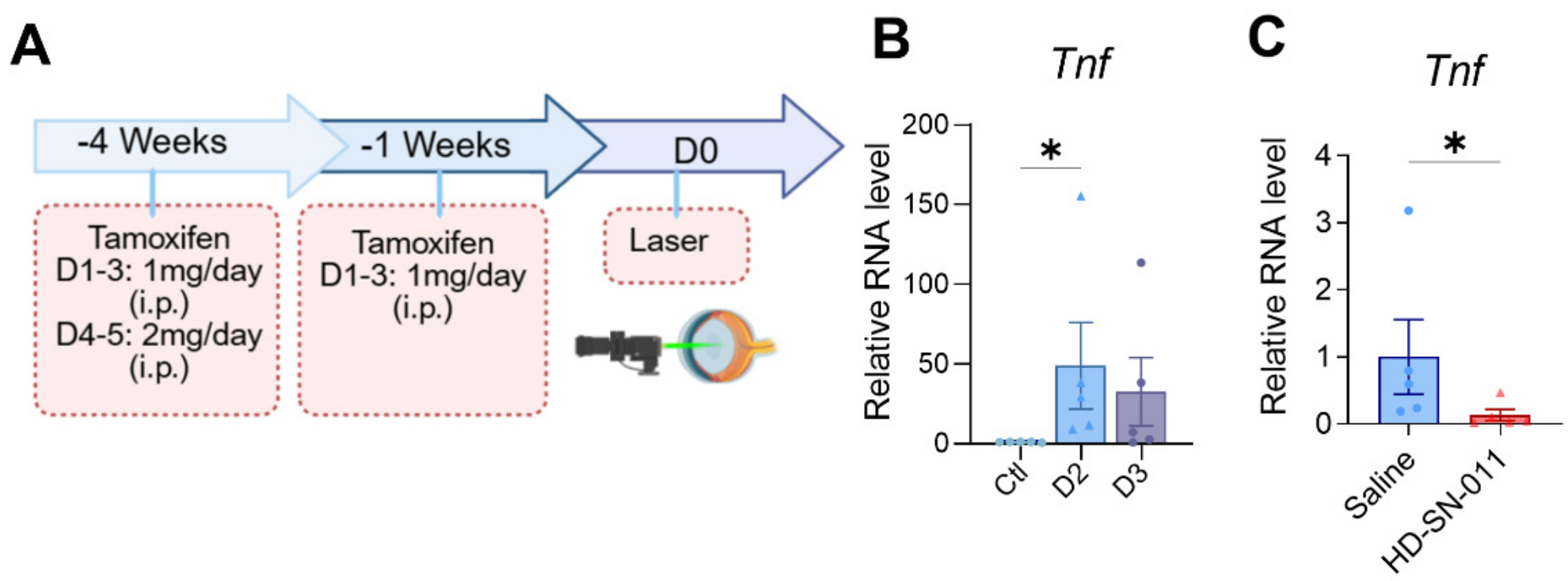

Supplementary Fig 5. HD-SN-011 attenuates MP cGAS–STING activation and downstream type I interferon–related inflammatory gene expression.

(A) Schematic of the tamoxifen administration schedule and laser induction in Cx3cr1-CreER; Rpl22$^{HA/HA}$ mice used for RiboTag-based MP translatome profiling. **(B)** qPCR analysis of TNF-α in HA-IP–enriched mRNAs at baseline (Ctl) and at Day 2 or Day 3 after laser-induced CNV in saline-treated eyes. **(C)** Mice subjected to laser-induced CNV received intravitreal injection of saline or HD-SN-011 on Day 1, followed by qPCR analysis of *Tnf* on Day 2. The saline group shown in (C) corresponds to the same day 2 control cohort shown in (B). Data are represented as mean ± SEM. Statistical significance was assessed by one-way ANOVA with Šidák's multiple comparisons test (B) or two-sided unpaired t-test (C). *$p < 0.05$.

Supplementary Table 1. Antibodies and Reagents

Antibody/Reagent	Company	Catalog
STING	Cell Signaling Technology	13647T
β-actin	Cell Signaling Technology	4970L
cGAS	Santa Cruz	sc-515777
P-TBK1	Cell Signaling Technology	5483T
TBK1	Cell Signaling Technology	3504T
P-IRF3	Cell Signaling Technology	29047T
IRF3	Abcam	ab68481 (IF)
IRF3	Cell Signaling Technology	4302T
IBA-1	FUJIFILM Wako Pure Chemical Corporation	019-19741
CD31	BD Biosciences	553370
TNF-α	Santa Cruz	sc-1350
IL-1β	Santa Cruz	sc-1252
2’3’ cGAMP	Invivogen	tlrl-nacga23-02
HA-IP beads	MBL	M-18011
Ethylenediamine-core PAMAM dendrimer (G4, 64-OH) (HD)	Dendritech Inc	2134
DMF (Dimethylformamide)	Sigma-Aldrich	227056-1L
DCM (Dichloromethane)	Sigma-Aldrich	270997-1L
TFA (Trifluoroacetic acid)	Sigma-Aldrich	8082600101
BOC-GABA-OH	Sigma-Aldrich	469572-25G

EDC (1-Ethyl-3-(3-dimethylaminopropyl)carbodiimide)	Sigma-Aldrich	03450-25G
DMAP (4-Dimethylaminopyridine)	Sigma-Aldrich	107700-25G
SN-011	MedChemExpress	HY-1450/CS-0312056
