# Supplementary material for: Mononuclear phagocyte–specific cGAS/STING targeting suppresses experimental choroidal neovascularization
Source: JCI Insight. 2026 Aug 10;11(15):e203452. doi: 10.1172/jci.insight.203452 (PMC13464012; doi:10.1172/jci.insight.203452)

Full unedited gel for Figure 1 B

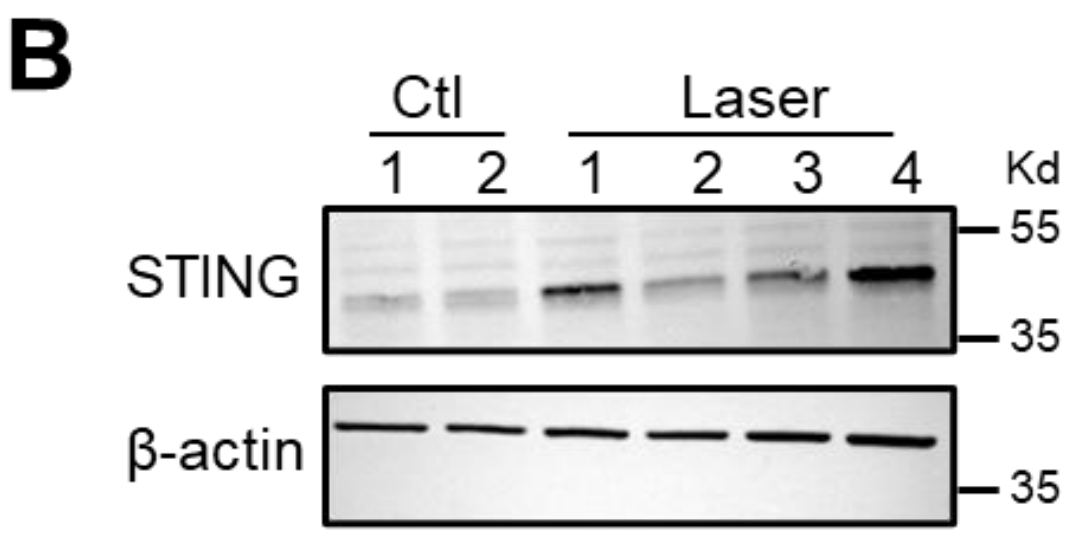

Figure 1B in the manuscript

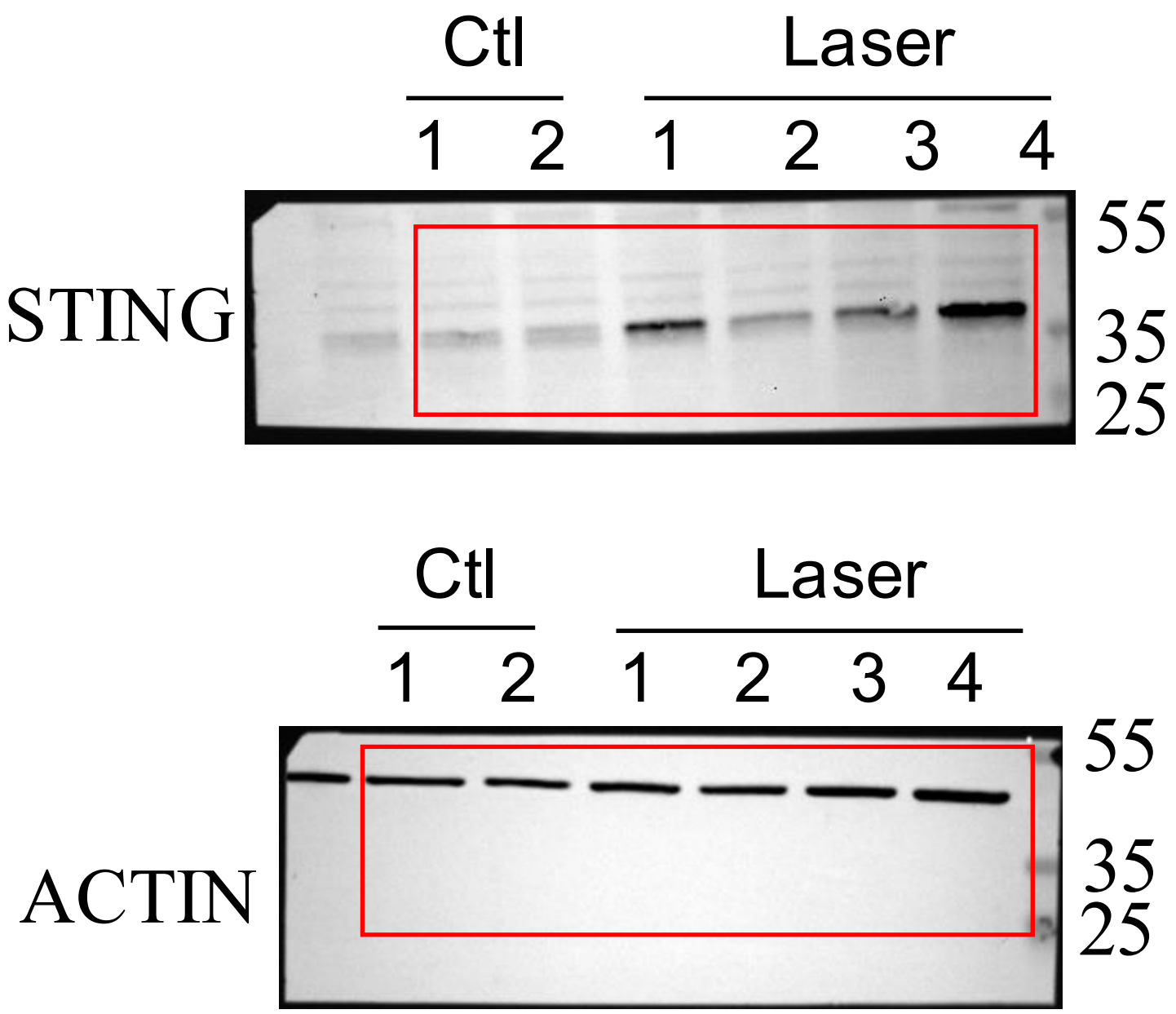

Full unedited gel for Figure 4F

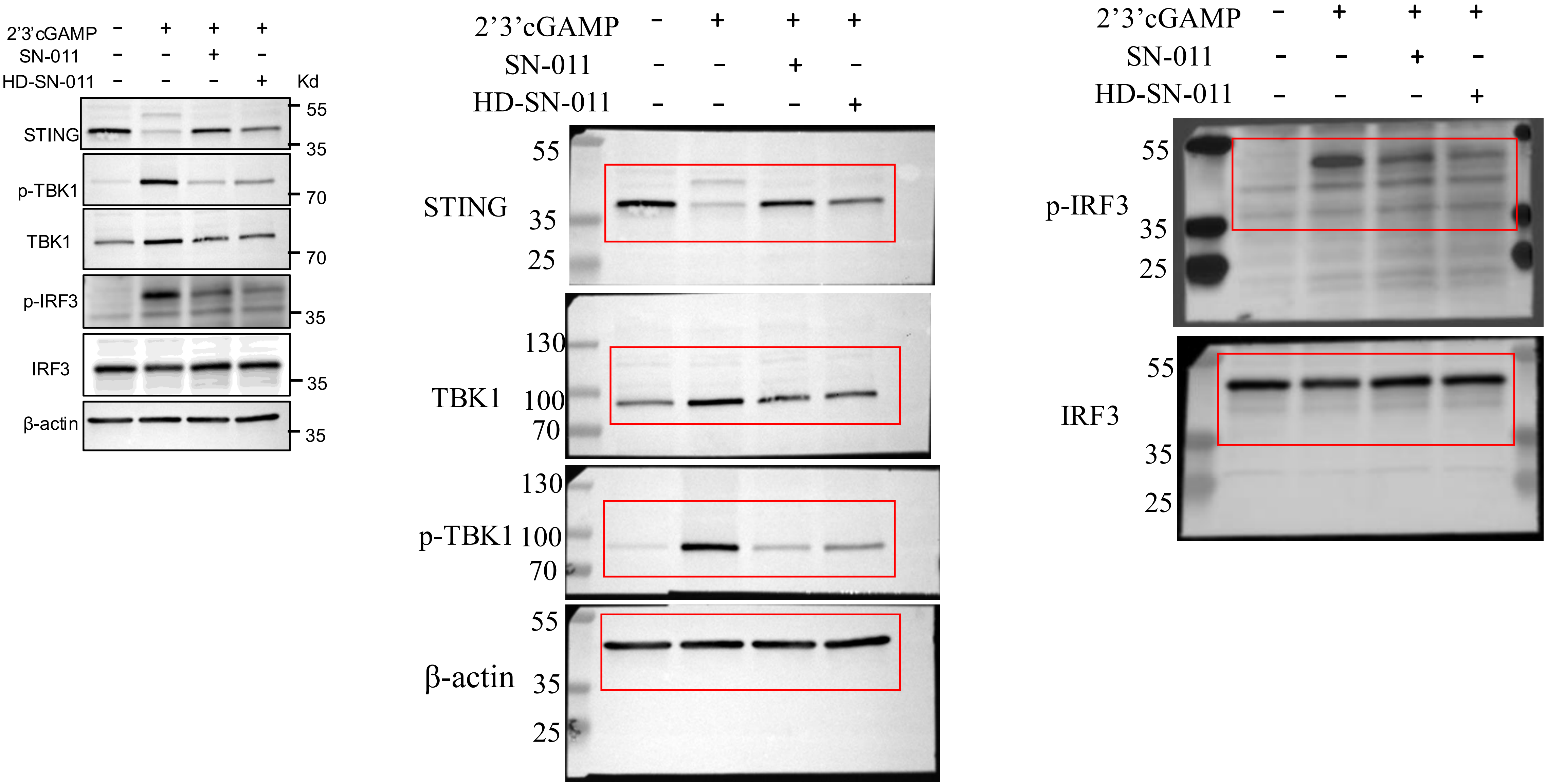

Supplement: Supplementary file 2 [file jciinsight-11-203452-s329.pdf]
